# Disturbance reshapes functional redundancy and accelerates nitrification in soil nitrifying communities

**DOI:** 10.1093/ismejo/wrag154

**Published:** 2026-06-16

**Authors:** Jun Zhao, Shannon M Brown, Jonathan Rodriguez, Eunkyung Choi, Nina Charissa A Infantado, Christina Hazard, Graeme W Nicol, Sarah L Strauss, Willm Martens-Habbena

**Affiliations:** Fort Lauderdale Research and Education Center, Department of Microbiology and Cell Science, University of Florida, Davie, FL 33314, United States; Fort Lauderdale Research and Education Center, Department of Microbiology and Cell Science, University of Florida, Davie, FL 33314, United States; Fort Lauderdale Research and Education Center, Department of Microbiology and Cell Science, University of Florida, Davie, FL 33314, United States; Fort Lauderdale Research and Education Center, Department of Microbiology and Cell Science, University of Florida, Davie, FL 33314, United States; Southwest Florida Research and Education Center, Department of Soil, Water and Ecosystem Sciences, University of Florida, Immokalee, FL 34142, United States; Laboratoire d’Ecologie Microbienne, Université Lyon 1, CNRS, INRAE, VetAgro Sup, Villeurbanne 69622, France; Laboratoire d’Ecologie Microbienne, Université Lyon 1, CNRS, INRAE, VetAgro Sup, Villeurbanne 69622, France; Southwest Florida Research and Education Center, Department of Soil, Water and Ecosystem Sciences, University of Florida, Immokalee, FL 34142, United States; Fort Lauderdale Research and Education Center, Department of Microbiology and Cell Science, University of Florida, Davie, FL 33314, United States

**Keywords:** environmental disturbance, functional redundancy, interspecific competition, nitrogen cycle, ammonia-oxidizing microorganisms, greenhouse gas emissions, soil microcosm, nitrification inhibitor

## Abstract

Statistical and culture-based models propose that environmental disturbances reshape competitive interactions among functionally redundant microbial taxa. However, the mechanisms driving these changes and their impact on biogeochemical processes remain largely untested in soil, owing to the challenge of linking functions to specific taxa in highly diverse and functionally complex soil microbiomes. Here, we simulated environmental disturbance in microcosms containing organic carbon-rich or sandy soils. Using bacterial and archaeal nitrifiers, a functionally tractable microbial guild, we examined how disturbance restructures competition among diverse microbial taxa within this guild. In both soils, ammonia-oxidizing archaea (AOA) predominated numerically and functionally under steady climax conditions. Following disturbance, ammonia-oxidizing bacteria (AOB) and complete ammonia oxidizers (comammox) rapidly gained a growth-related competitive advantage, likely due to increased per-cell ammonium availability supporting their intrinsic high growth rates. AOB recolonization was essential for full post-disturbance nitrogen turnover, resulting in elevated nitrification rates and increased nitrous oxide emissions. Nitrification rate did not fully recover when AOB were inhibited. In contrast, AOA and comammox played a dispensable role in recovering post-disturbance nitrification, limited by slower growth and lower per-cell activity, respectively. Competitive regrowth ability of microbial species showed a tradeoff with pre-disturbance abundance, highlighting the enhanced post-disturbance role of rare-abundance AOA, in addition to AOB phylotypes. Our findings demonstrate that bacterial and archaeal nitrifiers constitute a continuous spectrum between *r*- and *K*-strategists. Disturbance reshapes competition through differential growth and activity traits among functionally redundant taxa, favoring AOB and thereby transforming community assembly while intensifying nutrient cycling and greenhouse gas fluxes.

## Introduction

Global change intensifies the magnitude, frequency, and duration of environmental disturbances, such as extreme weather events, prolonged droughts, floods, and wildfires [1, 2], leading to microbial mortality and subsequent community reassembly [3]. Microorganisms exclusively drive many essential biogeochemical processes and play a central role in regulating nutrient transformations [4]. Although previous research has documented concurrent shifts in soil microbial community composition and nutrient pools following severe disturbances [5–7], establishing cause–effect relationships between community dynamics and ecosystem function in natural settings remains challenging. Given that microbial post-disturbance responses and growth rates often exhibit phylogenetic conservation [8–10], it is crucial, yet technically difficult *in situ*, to mechanistically characterize the different stages of secondary succession within functional microbial guilds before a quasi-stable state is reached.

Theoretical models suggest that physiological differences among functionally redundant microbial guilds drive asynchronous taxonomic responses to environmental disturbances, providing an ecological basis for maintenance, or even enhancement, of ecosystem functioning after disturbances [11–13]. Consequently, species’ roles and interactions are expected to shift as environmental conditions change [14]. Empirical evidence from synthetic culture experiments has shown that interspecific competition remains strong but is altered after disturbances [15], and that the outcomes of such competition are influenced by both the intensity of the disturbance as well as the initial population densities of the involved species [16]. As a result, microbial taxa that play a negligible role in a stable environment may thrive on newly available resources and become pivotal following environmental changes [17]. However, whether ecological theories derived from these modeling and culture-dependent studies extend to a complex soil ecosystem remains unclear, and the recolonization dynamics of functionally redundant taxa that impact ecosystem recovery are still poorly understood.

One common and critical environmental outcome of severe disturbances is the loss of reactive nitrogen (N) in soil [18–21]. Nitrification, a central step in the N cycle, accounts for one of the largest reactive N fluxes in the global N budget [22] and likely drives post-disturbance N loss through enhanced nitrate leaching and N_2_O emissions [21, 23–27]. The first step of nitrification, oxidation of ammonia to nitrite, is carried out by ammonia-oxidizing archaea (AOA) and ammonia-oxidizing bacteria (AOB), as well as complete ammonia oxidizers (comammox). Nitrite oxidizers, including nitrite-oxidizing bacteria (NOB) and comammox, complete the second step of nitrification by oxidizing nitrite to nitrate. Although different nitrifying phylotypes frequently coexist in soils, only a subset is typically active under specific environmental conditions, indicating substantial functional redundancy within nitrifier communities. Indeed, *Nitrosospira* AOB are redundantly distributed across many unfertilized soils, where a limited number of AOA taxa often dominate steady-state nitrification [28, 29]. These nitrifying groups comprise highly diverse species that exhibit varying kinetic properties, including substrate affinities, growth rates, and metabolic activities [30–33]. Such differences are expected to alter interspecific competitive interactions following environmental disturbance [14]. However, a recent study showed that the substrate affinity of soil AOA and AOB cultures overlap substantially [30], implying that they might respond similarly to environmental disturbance or other physiological traits may underlie their coexistence and competition.

AOB have been characterized as *r*-strategists, exhibiting primary activity in ammonium-enriched environments [34]. Consistently, rapid net AOB growth has been observed under conditions of substantially increased ammonium availability, such as following external ammonium-based fertilization at rates of 100 μg N g^−1^ soil or higher [35–37]. More recently, AOB growth has also been successfully stimulated in unfertilized soils (< 10 μg NH_4_^+^-N g^−1^ soil), but only when AOA growth was selectively inhibited [38], indicating that AOB can physiologically adapt to and maintain growth under much lower bulk ammonium concentrations than previously recognized. This observation suggests that enhanced AOB growth may depend not only on total ammonium concentration but also on ammonium availability at the individual-cell level in soil. Therefore, processes that reduce microbial abundance, thereby increasing ammonium availability per remaining cell without substantially altering total N pools, may also stimulate AOB growth.

Compared to AOB, AOA exhibit greater taxonomic diversity and encompass a wide range of life strategies and kinetic traits [30], including taxa that display kinetic characteristics overlapping with AOB, as well as some of the most oligotrophic nitrifying strains known. These characteristics likely contribute to their widespread distribution and ecological success in both nutrient-rich [28] and -limited environments [30]. However, among the 18 putative family-level AOA lineages characterized thus far, 11 still lack cultivated strains, and their kinetic properties remain unknown [39]. Within these yet-uncultivated taxa, many are ubiquitously present in soil, such as families of NS-delta and NS-beta, but have shown no detectable activity in soils under stable conditions [39–42]. These potentially dormant AOA taxa, which are predicted from comparative genomics to possess distinct nutrient acquisition strategies [40, 43], may gain a competitive advantage in disturbed soils, where nutrient availability is transiently redistributed.

Here, we investigate the secondary succession patterns of nitrifiers at high phylogenetic resolution and assess their associated ecological consequences following a severe disturbance event. We conducted a large-scale population reduction by inoculating 1% or 5% of fresh soil into gamma-irradiated soil in environmentally controlled soil microcosms. High levels of microbial mortality can occur following natural disturbances. For example, prolonged drought-rewetting events can reduce living microbial abundance by up to 70% across taxa due to osmotic shock [10, 44, 45], whereas wildfires typically result in an average loss of 60% and up to 96% in microbial biomass [46, 47]. Such an intensity of the imposed disturbance preserved most nitrifier diversity (Fig. S1), allowing us to uncover the competitive interactions among largely uncultured taxa governing succession dynamics and the functional recovery of nitrifying communities in response to severe disturbances. We hypothesize that (i) recovery of nitrification is associated with shifts in the competitive status of different soil ammonia-oxidizing species following disturbance, but the recolonization patterns of AOA and AOB partially overlap; (ii) AOB exhibit enhanced growth and activity due to an increased nutrient availability at the individual-cell level; and (iii) AOA display asynchronous recolonization dynamics at fine taxonomic resolution in soils, consistent with dissimilar life-history traits previously observed from culture-based experiments.

## Materials and methods

### Soil description, collection, and sterilization

Two physiochemically distinct soils were used for this study. One soil type was collected from a sugarcane plot in the Everglades Agricultural Area (EAA) near Belle Glade, Florida (26.65°N, 80.63°W) in July 2021 (for 95-day disturbance experiment) and August 2023 (for 30-day microcosm experiment). The soil derives from Pahokee and Terra Ceia muck, is classified as an euic, hyperthermic Lithic Haplosaprist (websoilsurvey.nrcs.usda.gov) and contains ~ 65% organic matter. The plot was used for sugarcane growth since 2012 and was not fertilized since May 2018, and a year-round biogeochemical and metagenomic survey between May 2017 and April 2018 demonstrated relatively stable *in situ* N transformation activity and microbial diversity in this soil plot, with AOA-predominating ammonia oxidation [28, 40]. The second soil type was collected from a sugarcane plot in Clewiston (CL), Florida (26°75°N, 80.96°W) in August 2023. The soil derives from Immokalee sand is classified as a sandy, siliceous, hyperthermic Arenic Alaquod (United States Department of Agriculture [USDA]), and contains ~1.5% organic matter. More details on the properties of the soils were described previously [28].

The top 10-cm surface soil was collected, homogenized, and sieved (1.8 mm mesh size) before use. A portion of the soil (~1.0 kg) was sterilized by gamma-irradiation (~50 kGy; ^137^Cs) to minimally disturb soil chemical properties [48] and the remaining fresh soil was stored at 4°C before microcosm construction. Tests by quantitative polymerase chain reaction (qPCR) and net nitrification incubations confirmed that archaeal and bacterial *amoA* genes, and bacterial *nxrB* genes, as well as nitrification activity were below detection limit after irradiation.

### Simulation of population-reduction disturbance in microcosm

Soil was used to establish post-disturbance microcosm experiments. Irradiation did not affect soil moisture. Therefore, the fresh soils were acclimated to ambient temperature for 7 days, and then mixed thoroughly with the sterilized soil to simulate the consequence of population-reduction disturbance. For EAA and CL soil, the irradiated soil and fresh soil were mixed at ratio of 95:5 (5% fresh soil inoculation) and 99:1 (1% fresh soil inoculation), respectively. These inoculation levels reflect the consequences of some extreme disturbance under natural conditions [47]. The higher proportion of irradiated soil in the low-organic matter CL treatment was intended to provide more available nutrients for surviving microbes, enabling more sensitive detection of recolonization by growth rate-based approach. In all microcosms, soil moisture was maintained at 53% for EAA soil and 10% for CL soil (corresponding to ~ 30% and ~ 40% water-filled pore space), respectively, which approximates field water content and favors aerobic nitrification activity [49].

For EAA soil microcosm experiments, triplicate soil microcosms were constructed in 250 ml jars each containing 40 g of the mixed soil. The mixed soil was amended with sterile water (treatment “disturbed-H_2_O”) for a period of 95 days incubation at 28°C in darkness. Undiluted fresh soil, homogenized and incubated under the same conditions, was used as a control (treatment “undisturbed-H_2_O,” with two replicates; the third replicate bottle broke during the experiment). To assess whether additional N supply would affect the trajectory of microbial secondary succession, additional disturbed soil treatments were established by amending with ammonium chloride or urea solution at 50 μg N g^−1^ dry weight soil at 3- to 4-day intervals (treatment “disturbed-NH_4_Cl” and “disturbed-urea”). The N compounds were dissolved in 100 μl of solution and applied accordingly, with additional water added as needed to maintain soil moisture at ~ 53%. After measuring the CO_2_ and N_2_O concentrations in the headspace of the microcosms, 0.5 g of soil was non-destructively collected from each microcosm at Days 0, 5, 7, 8, 11, 13, 15, 19, 23, 27, 35, 47, 59, 71, 83, and 95 for determination of ammonium, nitrite, and nitrate concentrations. For molecular analysis, 0.25 g soil was collected non-destructively at Days 0, 7, 15, 23, 35, 47, 59, 71, 83, and 95. An additional microcosm treatment containing only sterilized soil was also established to calibrate the gas emission from the disturbed soil.

Based on the results from the extended 95-day experiment, we shortened the follow-up microcosm incubation period to 30 days (including the selective inhibition experiments) and included CL soils. The microcosms were established in 120 ml jars each containing 10 g of the mixed soil, and incubated under the same conditions. Soil of 0.5 g was non-destructively sampled at Days 0, 6, 12, 18, 24, and 30 for determination of ammonium, nitrite, and nitrate concentration. Soil of 0.25 g was collected at Days 12 and 30 for DNA extraction and molecular analysis.

### Selective inhibition experiments on disturbed soil microcosms

To evaluate the potential contributions of different nitrifying groups in the disturbed soil microcosms, five different inhibitors with a series of concentrations were tested, including dicyandiamide (DCD), allylthiourea (ATU), potassium chlorate (KClO_3_), 1-octyne, and simvastatin (SVS). Each inhibitor targets specific groups of nitrifiers, and the applied concentrations were selected based on previous studies on their potency and specificity: ATU was supplied at rates of 6 μg–6 mg g^−1^ soil; DCD was applied at rates of 5 μg–5 mg g^−1^ soil; KClO_3_ was applied at rates of 3 μg–3 mg g^−1^ soil; 1-octyne was applied at a headspace concentration of 0.003%–0.3% (v/v); and SVS was amended in the soil at the rate of 0.2–20 mg g^−1^ soil (see details in Supplementary methods). Disturbed microcosms for EAA and CL soils were incubated in darkness at 28°C for 30 days, with non-destructive sampling performed as described above.

### Nutrient analysis and gas measurement

Soil inorganic N was extracted by mixing 0.5 g soil with 1.5 ml of 2.0 M KCl solution for 1.0 h followed by centrifugation at 5000 *g* for 5 min. The supernatant was used to determine ammonium (NH_4_^+^), nitrite (NO_2_^−^), and nitrate (NO_3_^−^) concentrations colorimetrically as previously described [28]. Soil pH was measured using 5.0 g soil in a soil to water ratio of 1:2 (w/w). Nitrification potential was measured as previously described [28] using both undisturbed and disturbed microcosm soil after 95-day incubation. CO_2_ and N_2_O concentrations were measured by collecting 5 ml of headspace gas and injecting into a gas chromatograph with methanizer-FID and ECD detectors (SRI Instruments, Las Vegas, NV) as previously described [28].

### DNA extraction and quantification of *amoA* and *nxrB* genes

Soil DNA was extracted using 0.25 g of the soil by DNeasy PowerSoil Pro Kit (QIAGEN, Germantown, MD) as previously described [40], and was diluted 20 times to < 10 ng μl^−1^ for PCR analysis. The *amoA* gene abundances of AOA, AOB, and comammox were determined using primer sets amoA23f/amoA616r [50], amoA1F/amoA2R [51], and Ntsp-amoA 162F/359R [52], respectively. Canonical *Nitrospira nxrB* gene abundance was quantified using primer nxrB169f/nxrB638r [53]. qPCR was performed on a Bio-Rad IQ5 real-time PCR system (Bio-Rad, Hercules, CA), following the reaction conditions of each gene detailed in Table S1. Genomic DNA extracted from pure cultures of strains *Nitrososphaera viennensis* EN76, *Nitrosospira multiformis* ATCC 25196, Ca. *Nitrospira inopinata* ENR4, and *Nitrospira moscoviensis* NSP M-1 were used to prepare qPCR standards for AOA, AOB, comammox *amoA*, and *Nitrospira nxrB* genes, respectively, ranging from 10^1^ to 10^6^ gene copies per reaction. The efficiencies of qPCR assays for AOA, AOB, comammox *amoA*, and *Nitrospira nxrB* were 95.5%–99.2%, 92.4%–99.4%, 76.9%–79.3%, and 79.6%–86.2%, respectively, with all *R*^2^ values > 0.99.

### Amplicon-sequencing and analysis of *amoA* and *nxrB* genes

For the 95-day EAA soil microcosms, archaeal *amoA* genes from microcosms at Days 0, 7, 15, 23, 47, 71, and 95 were amplified for amplicon sequencing using the same primer sets for qPCR assays, after incorporating adapters. Because AOB and comammox abundances reached the peak at Day 7 and NOB abundance peaked at Day 15 in the disturbed microcosms, amplicon-based sequencing was performed for bacterial and comammox *amoA* genes at Days 0, 7, and 95, and for *nxrB* gene at Days 0, 15, and 95. The library construction and sequencing of the archaeal *amoA* amplicons (2 × 300 bp) were performed on the MiSeq platform (Illumina, San Diego, CA). For bacterial and comammox *amoA*, and *nxrB* gene amplicons, the sequencing (2 × 250 bp) was performed on the NovaSeq 6000 platform (Illumina) following standard protocols by Novogene, California, United States. For 30-day EAA and CL soil microcosms in the absence or presence of inhibitors, all amplicon sequencing (2 × 250 bp) was performed on the NovaSeq 6000 platform.

Because the amplified archaeal *amoA*, bacterial *amoA*, and *nxrB* genes were 629, 491, and 485 bp in length, respectively, the paired-end reads could not be merged. Therefore, the raw sequences were processed following the previously established “gapped” pipeline for analyzing functional gene sequences [54], with a few modifications (see details in Supplementary methods). Because the amplicon length of comammox *amoA* gene was 198 bp, only forward sequences were used for bioinformatic analysis. Final high-quality reads were subsequently subjected to de novo clustering at 99% (for *amoA* genes) or 80% (*nxrB* gene) sequence identity to generate relative abundance of operational taxonomic units (OTUs) using VSEARCH [55]. Representative reads were assigned to different nitrifier lineages using BLAST+ tool against curated AOA [39], AOB [54], comammox *amoA* (https://github.com/miasungeunlee/AMOA-SEQ), or bacterial *nxrB* [53] gene reference sequence databases (query coverage > 90%, identity > 80%, e-value < 10^−5^), after deleting the same “gapped” region in the reference database sequences. The abundance of OTUs belonging to the same nitrifier lineage was summed to calculate its proportion relative to the total community.

### Statistical analysis

The effect of incubation time and treatment on the log_10_-tranformed *amoA* or *nxrB* gene abundance of each nitrifier group (AOA, AOB, comammox, or NOB) and the concentration of different forms of N (ammonium, nitrite, or nitrate) was tested by a factorial two-way ANOVA, with Tukey’s HSD test to assess the significant difference (*P* < .05) in SPSS 23.0.

Taxon-specific microbial abundances were calculated as described previously [10, 56]. Briefly, absolute abundance of each family-level AOA lineage in the soil was calculated individually for each microcosm as: lineage X abundance = total AOA abundance × lineage X proportion. The “lineage X abundance” is the absolute abundance of lineage X in a microcosm replicate presented as g^−1^ dry weight soil; the “total AOA abundance” was quantified by qPCR of archaeal *amoA* gene, presented as g^−1^ dry weight soil; and “lineage X proportion” is the percentage of lineage X read number relative to the total archaeal *amoA* read number derived from amplicon high-throughput sequencing analysis described above. Absolute abundance was log_10_-transformed and the temporal abundance pattern of each AOA lineage in different microcosm treatments was plotted using locally weighted polynomial regression with a LOESS curve fitting procedure. A two-way ANOVA was performed on log_10_-transformed abundances to test the effect of incubation time and treatment on the abundance of each nitrifying lineage. To assess the change in abundance of ammonia-oxidizing taxa at finer taxonomic resolution (species/strain level), absolute abundances of ammonia-oxidizing *amoA* OTUs were calculated and statistically analyzed using the similar method described above, after removal of rare OTUs with maximum relative abundances < 1.0% of total read number in any soil sample.

The minimal doubling time was calculated for each AOA lineage based on the temporal increase in abundance during exponential growth phase in all disturbed soil microcosms. The doubling time of an AOA family was calculated separately for each replicate microcosm from at least three time points during the incubation prior to statistical analysis. However, for a few AOA families in some microcosms (11% of the result), coefficient of determination was low using three time points (*R*^2^ < 0.8), indicating the maximum growth rate was only detected over a relatively short period of time. In those cases, two time points were used to calculate the minimal doubling time. One-way ANOVA was used to assess whether the doubling time was different between different AOA lineages in the disturbed soil microcosms, with Tukey’s HSD test to assess the significant difference. Similarly, the minimal doubling time of each AOA OTU was calculated using three time points (*R*^2^ > 0.7), whereas for AOB and comammox OTUs, only two time points (Days 0 and 7) were used, as their abundance peaked by Day 7. Multiple-comparison corrections were consistently applied in all ANOVA tests.

The effectiveness of an inhibitor on a specific nitrifying community in disturbed soil was evaluated by measuring the community’s growth in the presence of the inhibitor relative to its growth in the absence of any inhibitor. This was calculated using the formula:


$$\mathrm{Relative}\ \mathrm{growth}=\frac{\mathrm{I}X-\mathrm{D}0}{\mathrm{C}X-\mathrm{D}0}$$

where “I*X*” represents gene abundance on Day *X* (Day 12 or 30) in the presence of the inhibitor, “C*X*” represents gene abundance on Day *X* in the control microcosms without any inhibitor, and “D0” represents gene abundance in the disturbed microcosms at Day 0. If the value of relative growth is ≤ 0, it suggests complete inhibition by the inhibitor; a value ≥ 1 indicates non-inhibition or promotion of growth by the inhibitor; and a value between 0 and 1 indicates partial inhibition. The statistical significance of growth inhibition or promotion was determined by comparing gene abundance in disturbed microcosms with and without the inhibitor at the same sampling point using a *t-*test.

## Results

### Disturbance enhances competitive growth of bacterial ammonia oxidizers and accelerates nitrogen turnover

**Figure 1:**
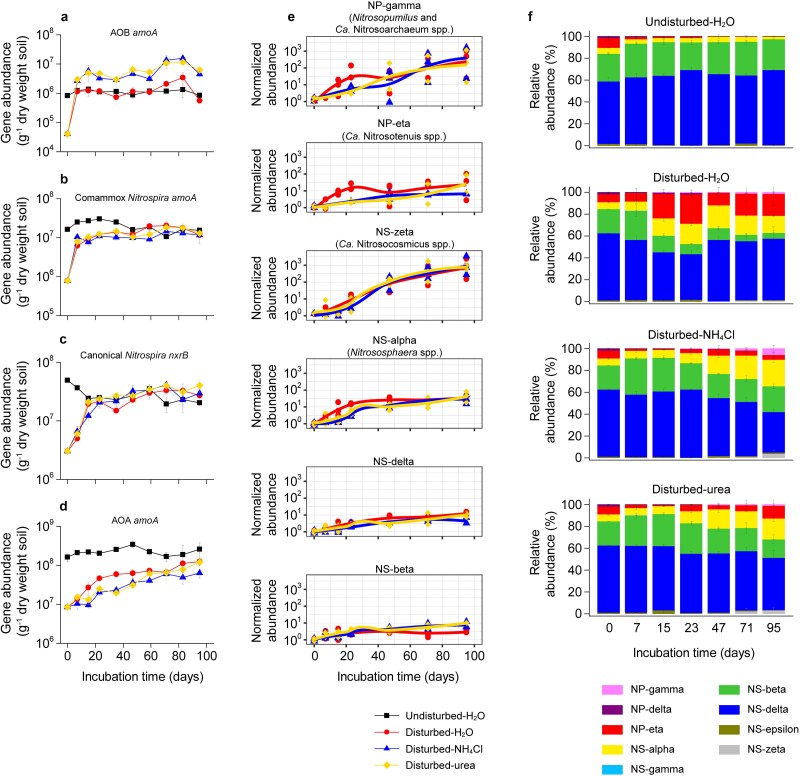
Post-disturbance recolonization of different nitrifying taxa. Microcosms contained unsterilized soils amended with water (“undisturbed-H_2_O”) or sterilized soils mixed with 5% (w/w) of fresh soil following amendment with water (“disturbed-H_2_O”), ammonium chloride (“disturbed-NH_4_Cl”), or urea (“disturbed-urea”). (a–d) Ammonia-oxidizing bacteria (AOB), complete ammonia-oxidizing *Nitrospira* (comammox), canonical nitrite-oxidizing *Nitrospira* (NOB), and ammonia-oxidizing archaea (AOA) were quantified based on *amoA* or *nxrB* genes by real-time quantitative PCR. Data represent mean values and standard errors of three replicate microcosms (two replicates for “undisturbed-H_2_O” with error bars representing standard deviations). (e) The abundance of specific AOA lineages in a disturbed soil was calculated by multiplying the total AOA abundance and the relative abundance of the lineage. To compare the fold-change growth, the abundance of each AOA lineage was normalized by the abundance at Day 0. Data were plotted using locally weighted polynomial regression with a LOESS fitting curve from three replicate microcosms. The species name is shown in the parentheses if a pure strain in the corresponding lineage has been cultivated. (f) Change in the proportion of different AOA lineages relative to total AOA abundance over the microcosm incubation. Data represent mean values and standard errors of three replicate microcosms (two replicates for “undisturbed-H_2_O” with error bars representing standard deviations).

The abundances of all nitrifiers remained unchanged over 95-day incubation in the undisturbed, organic-rich EAA soil microcosms (treatment “undisturbed-H_2_O,” *P* > .05; Fig. 1a–d), indicating a stable ecological state of soil nitrifiers without major incubation artifacts. Under this condition, AOA outcompeted AOB and comammox in activity, corroborating prior activity-based and metatranscriptomic studies [28, 40].

**Figure 2:**
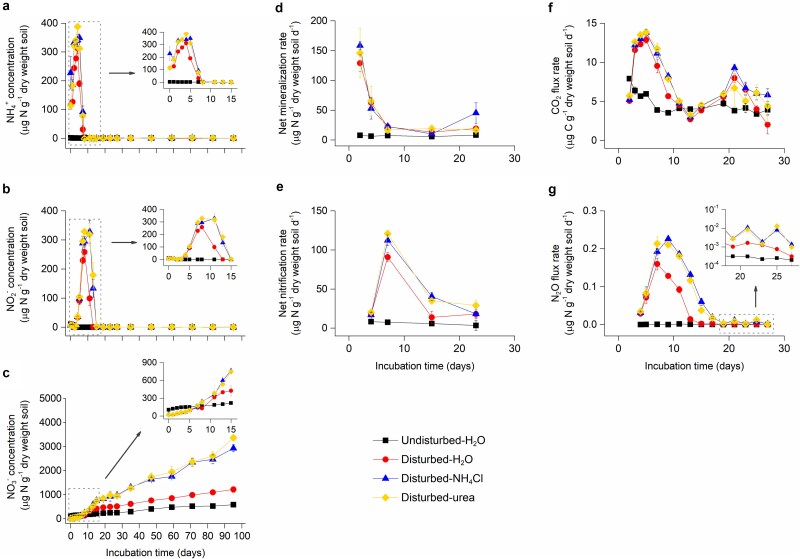
Accelerated post-disturbance nitrogen and carbon turnover. (a–g) Temporal changes in inorganic nitrogen (N) concentrations, N mineralization, nitrification, and CO_2_ and N_2_O production rates in microcosms. Microcosms contained fresh EAA soils amended with water (“undisturbed-H_2_O”) or sterilized soils mixed with 5% (w/w) of fresh soil following timely amendment with water (“disturbed-H_2_O”), ammonium chloride (“disturbed-NH_4_Cl”), or urea (“disturbed-urea”). (a–c) Soil NH_4_^+^, NO_2_^−^, and NO_3_^−^ concentrations in soil microcosms were measured during a 95-day incubation. (d and e) N mineralization and nitrification were measured for 23 days. (f and g) CO_2_ and N_2_O emission rates were measured for 27 days. Data represent mean values and standard errors of three replicate microcosms (two replicates for “undisturbed-H_2_O” soil microcosms with error bars representing standard deviations). Some error bars are smaller than the mean value symbol due to small variation between replications.

Disturbance triggered two distinct phases of taxon-specific microbial secondary successions associated with N transformations in this EAA soil in the 95-day incubation (treatment “disturbed-H_2_O”). The initial phase (“pulse phase”; Days 1–15) was characterized by rapid regrowth of bacterial ammonia oxidizers (AOB and comammox) and canonical nitrite oxidizers (NOB), which fully regained their pre-disturbance abundance within 7 and 15 days, respectively (Fig. 1a–c). In contrast, AOA showed a slower and only partial recovery during this stage (Fig. 1d). This suggests a shift in predominantly active ammonia oxidizers from archaea in undisturbed soil to bacteria in post-disturbance soil. Concurrently, this phase exhibited sequential pulses of ammonium (Days 1–4, peaking at 313 ± 19 μg N g^−1^ dry weight soil; Fig. 2a), nitrite (Days 4–8, peaking at 258 ± 17 μg N g^−1^ dry weight soil; Fig. 2b), and nitrate (Days 8–15; Fig. 2c). The highest post-disturbance net N mineralization (129.1 ± 14.4 μg N g^−1^ dry weight soil d^−1^) and nitrification (90.9 ± 5.8 μg N g^−1^ dry weight soil d^−1^) rates occurred during this stage, representing 16.1- and 10.8-fold increases, respectively, compared to undisturbed soil (Fig. 2d and e). Correspondingly, the associated CO_2_ and N_2_O production rates were significantly higher in disturbed than in undisturbed soil (Fig. 2f and g), leading to increased cumulative greenhouse gas emissions (Fig. S2). The N_2_O yield (i.e. N_2_O-N per NO*_x_*^−^-N produced) significantly increased from ~ 0.1‰ in the undisturbed soil microcosms to between 1.3‰ and 1.5‰ in the disturbed soil (Fig. S2), indicative of a shift from dominant AOA activity in the undisturbed soil to AOB in the disturbed soils [57].

The subsequent phase (“stabilization phase”; Days 15–95) was characterized by the slow, continuous growth of AOA. By Day 71, AOA abundance was no longer significantly different from that in undisturbed soil (*P* > .05; Fig. 1d). In contrast, the abundance of all bacterial nitrifier groups remained stable and reached a plateau (Fig. 1a–c). This resulted in a steady but lower post-disturbance nitrification rate at this stage (10.0 ± 0.9 μg N g^−1^ dry weight soil d^−1^), which was still 2.0-fold higher than in the undisturbed soil. Sustained nitrification led to a steady accumulation of nitrate (maximum of 1219 ± 118 μg N g^−1^ dry soil), without transient ammonium or nitrite accumulation (Fig. 2a–c). The NO*_x_*^−^ production was significantly positively correlated only with AOA abundance during this phase (Fig. S3). These findings suggest that AOA gradually regained their ecological significance in driving and stabilizing ammonia oxidation during this phase of secondary succession, with per-cell activities comparable across taxa.

The two distinct phases of microbial secondary succession were consistent even with additional N application (treatments “disturbed-NH_4_Cl” and “disturbed-urea”). Despite N fertilization, the abundance of all four nitrifying groups recovered on a similar timeline, with bacterial nitrifiers growing faster than archaea (Fig. 1a–d). The post-disturbance growth of most nitrifying groups remained unaffected by N fertilization after 95 days (Fig. 1b–d), except for increased net growth of AOB abundance following N supply (*P* < .05; Fig. 1a). Similarly, N fertilization produced consistent temporal patterns of inorganic N accumulation and greenhouse gas emissions in disturbed soil, except for a significantly higher nitrate accumulation peak (3150 ± 125 μg N g^−1^ dry soil) resulting from the conversion of externally supplied ammonium (Fig. 2).

Similar post-disturbance recolonization patterns were also found in the physiochemically contrasting sandy CL soil over a 30-day microcosm incubation. Although transient peaks in ammonium and nitrite accumulation were insignificant, likely due to a much lower organic matter and N turnover than in EAA soil, similar patterns emerged, including: (i) a faster recovery of bacterial nitrifiers compared to archaeal nitrifiers (Fig. S4) and (ii) significantly higher nitrate production following disturbance (63.7 ± 3.4 μg N g^−1^ dry soil) compared to undisturbed microcosms (25.1 ± 4.8 μg N g^−1^ dry soil).

### Competitive regrowth of ammonia oxidizers is inverse-density dependent and taxon-specific

Despite full recovery of nitrifier abundances, the community compositions of AOA, comammox, and NOB shifted during post-disturbance recolonization in EAA soil (Fig. S5). Of the 18 globally recognized AOA families [39, 41], we detected nine in the EAA soil, including all known *Nitrososphaerales*-affiliated families (NS-alpha, NS-beta, NS-gamma, NS-delta, NS-epsilon, and NS-zeta) and three *Nitrosopumilales* families (NP-gamma, NP-delta, and NP-eta). Six of these lineages exhibited significant growth (Fig. 1e; Fig. S6). Among them, NP-gamma (minimum doubling time of 2.5–3.8 days in all disturbed soil microcosms), NP-eta (2.3–4.5 days), NS-zeta (4.4–5.5 days), and NS-alpha (5.7–11.1 days) displayed significantly higher maximum growth rates than NS-delta (12.0–16.7 days) and NS-beta (12.9–18.9 days) (Table S2). In addition, the “magnitude of population recovery,” defined as fold-increase in cell abundance compared to Day 0, was highest for NS-zeta (1613.6- to 4144.8-fold increase), followed by NP-gamma (316.5- to 833.3-fold), NS-alpha (33.5- to 51.7-fold), NP-eta (6.6- to 50.5-fold), NS-delta (6.9- to 12.7-fold), and NS-beta (4.3- to 9.9-fold). These variations in regrowth patterns among AOA families contributed to a gradual yet significant shift in community composition over the incubation of disturbed EAA soil (Fig. 1f).

**Figure 3:**
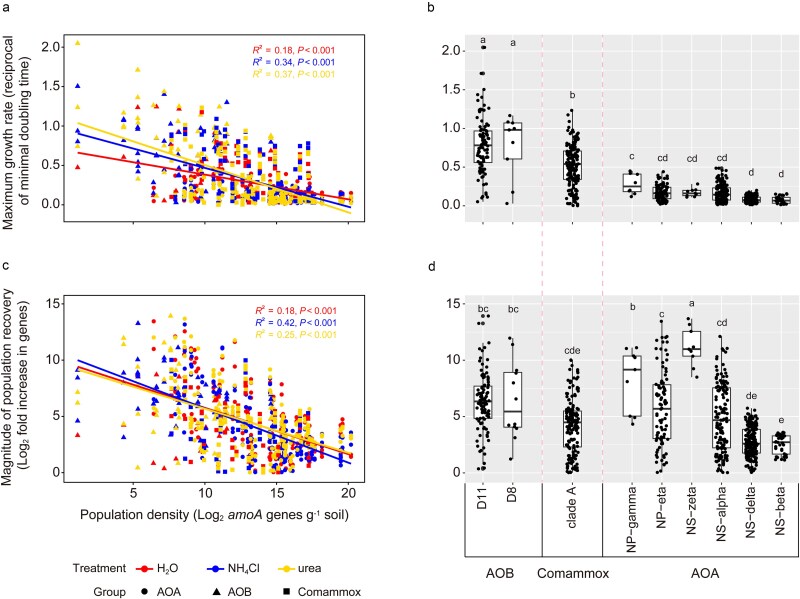
Population density-dependent post-disturbance regrowth of different nitrifying phylotypes. (a) Correlations between pre-disturbance population density and post-disturbance maximum growth rate of major ammonia-oxidizing OTUs during the incubations in EAA soil. The maximum growth rate was calculated as the reciprocal values of minimal doubling time over three time points, (b) Box plot showing the post-disturbance maximum growth rates of ammonia-oxidizing OTUs affiliated into broader taxonomic lineages. (c) The correlations between pre-disturbance population density and magnitude of population recovery across major ammonia-oxidizing OTUs after 95-day incubations. The magnitude of population recovery refers to the fold-increase in absolute *amoA* gene abundance over 95-day post-disturbance incubations. (d) Box plot showing the magnitude of population recovery of ammonia-oxidizing OTUs affiliated into broader taxonomic lineages. (a and c) Linear regression line was plotted from ammonia-oxidizing OTUs (AOA, AOB, and comammox) in different treatments (H_2_O, NH_4_Cl, and urea), with coefficient of determination (*R*^2^ value) and degree of significance (*P* value) displayed. (b and d) Different letters above the boxes indicate significant difference in growth rate or ratio between different ammonia-oxidizing lineages (*P* < .05).

At the species level, we established correlations between microbial competitive regrowth and the pre-disturbance population size of ammonia oxidizer phylotypes (OTUs with relative abundances exceeding 1.0% in at least one EAA soil microcosm, including 61 AOA, 34 AOB, and 27 comammox OTUs). Phylotypes with lower pre-disturbance population sizes exhibited higher maximum growth rates (i.e. shorter minimal doubling times, *P* < 0.001; Fig. 3a), consistent with the broad community recolonization pattern. Specifically, phylotypes of AOB and comammox showed significantly higher maximum growth rates than all AOA phylotypes (Fig. 3b). Similarly, the magnitude of population recovery also increased as pre-disturbance population size decreased across different phylotypes (*P* < .001; Fig. 3c). However, unlike growth rate, population recovery did not show community-level distinctions but was instead clustered at the finer taxonomic level (Fig. 3d). For example, at the family level, AOA phylotypes from NS-zeta, NP-gamma, and NP-eta exhibited significantly higher magnitude of population recovery than those from NS-delta and NS-beta. Similar species recolonization patterns were observed also in CL soil, including: (i) an inverse population density-dependent growth (Fig. S7); (ii) no clear community-level distinctions in population recovery (Fig. S7); (iii) family-level differences in AOA population recovery, where NS-delta and NS-beta species had significantly lower ratios than NS-zeta species (Fig. S7); and (iv) significant shifts in ammonia-oxidizing community compositions following disturbance (Fig. S5). Collectively, these findings indicate that ammonia-oxidizing species with lower pre-disturbance abundance grew more actively than higher-abundance species during the recolonization, suggesting increased contributions of initially low-abundance phylotypes to post-disturbance nitrification. Such negative density-dependent regrowth suggests shifted microbial competitive interactions during secondary succession [10].

### Competition influences post-disturbance community reassembly and N turnover rates

**Figure 4:**
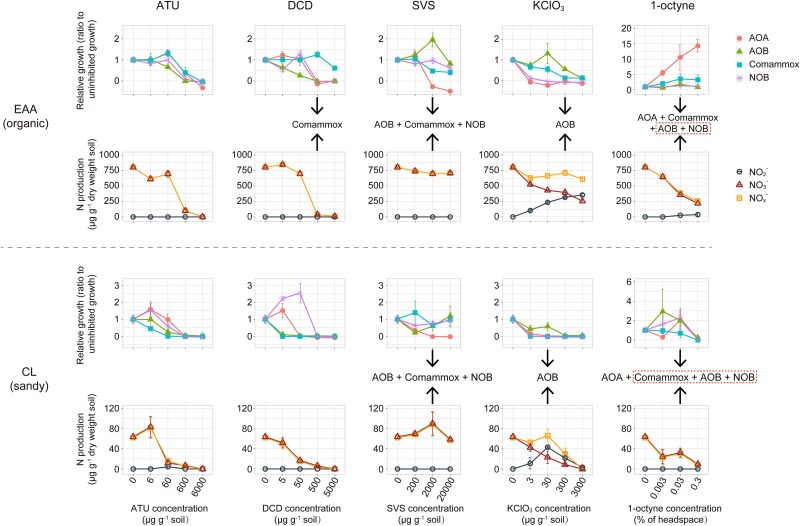
Effect of competition on the post-disturbance regrowth and activity of nitrifying communities. Competing nitrifying groups were modified using five selective nitrifier inhibitors followed by 30 days of microcosm incubation. The *y*-axis of relative growth represents the growth of the nitrifying community in response to a specific inhibitor relative to its growth in the absence of inhibitors. A value ≤ 0 indicates complete inhibition, a value ≥ 1 indicates no inhibition or potential promotion of growth, and a value between 0 and 1 indicates partial inhibition. The *y*-axis of N production represents the increase in nitrogen concentration relative to Day 0, measured as nitrite (NO_2_^−^), nitrate (NO_3_^−^), or their combined forms (NO*_x_*^−^). The *x*-axis represents the inhibitor concentration. Arrows indicate nitrifying communities that were not inhibited at a specific inhibitor concentration and contributed to the nitrogen turnover. The nitrifying groups in the dotted box were temporally inhibited by 1-octyne. Abbreviations: ATU, allylthiourea; DCD, dicyandiamide; SVS, simvastatin.

To verify competitive interactions between different nitrifying lineages, we performed a top-down manipulation experiment using selective inhibitors targeting specific nitrifying groups in both EAA and CL soils. After assessing the potency and efficiency of five distinct inhibitors at a series of concentrations (see details in Supplementary results and Figs S8–S12), we assessed the population growth of each ammonia-oxidizing group in follow-up 30-day post-disturbance microcosm incubations. In EAA soil, temporal inhibition of AOB by 1-octyne resulted in a 10.6- and 3.4-fold increase in the growth rate of AOA (*P* < .05 at Day 30) and comammox (*P* < .05 at Day 12; Fig. 4; Fig. S12). In CL soil, inhibition of AOB and comammox by 1-octyne led to tentative 2.0-fold increase in AOA growth, though the variation among replicates prevented statistical significance (Fig. 4). In addition, in EAA soil, AOB growth increased by 2.0-fold (*P* < .05 at Day 30) following inhibition of AOA by simvastatin. Together, these results indicate direct growth competition among the three ammonia-oxidizing groups during secondary successions.

Post-disturbance nitrification dynamics varied depending on the competing lineages present (Fig. 4). In both soils, the removal of AOA and comammox competition had no impact on nitrification dynamics, and AOB recolonization alone sustained post-disturbance ammonia oxidation rates equivalent to those achieved by all three ammonia-oxidizing groups combined (KClO_3_ inhibition of all other nitrifiers; Fig. 4; Fig. S11). However, the inhibition of AOB recolonization led to impaired post-disturbance nitrification activity over the incubation period (Fig. 4). In EAA soil, AOA and comammox combined accounted for no more than 47.8% ± 1.9% of NO*_x_*^−^ production when AOB were inhibited by 1-octyne, relative to uninhibited soil microcosms. Furthermore, comammox alone contributed only 4.8% ± 1.2% of post-disturbance NO*_x_*^−^ production when both AOA and AOB were inhibited by dicyandiamide. Similarly, in CL soil, AOA contributed at most 49.7% ± 14.2% of NO*_x_*^−^ production when AOB and comammox were inhibited by 1-octyne, compared to uninhibited conditions. Estimated potential cell-specific rates of AOB (365.6 ± 33.5 and 217.4 ± 163.2 fmol N cell^−1^ h^−1^ in EAA and CL soils, respectively) were orders of magnitude higher than those of AOA (0.145 ± 0.048 and 0.102 ± 0.021 fmol N cell^−1^ h^−1^ in EAA and CL, respectively) and comammox (0.330 ± 0.095 fmol N cell^−1^ h^−1^ in EAA). These findings suggest that neither AOA nor comammox, individually or together, can sustain the enhanced post-disturbance nitrification rates in the absence of AOB activity. Therefore, despite competitive interactions, AOB growth was integral to the post-disturbance ecosystem functioning, whereas AOA and comammox functioned as auxiliary lineages, complementing AOB activity during secondary succession.

**Figure 5:**
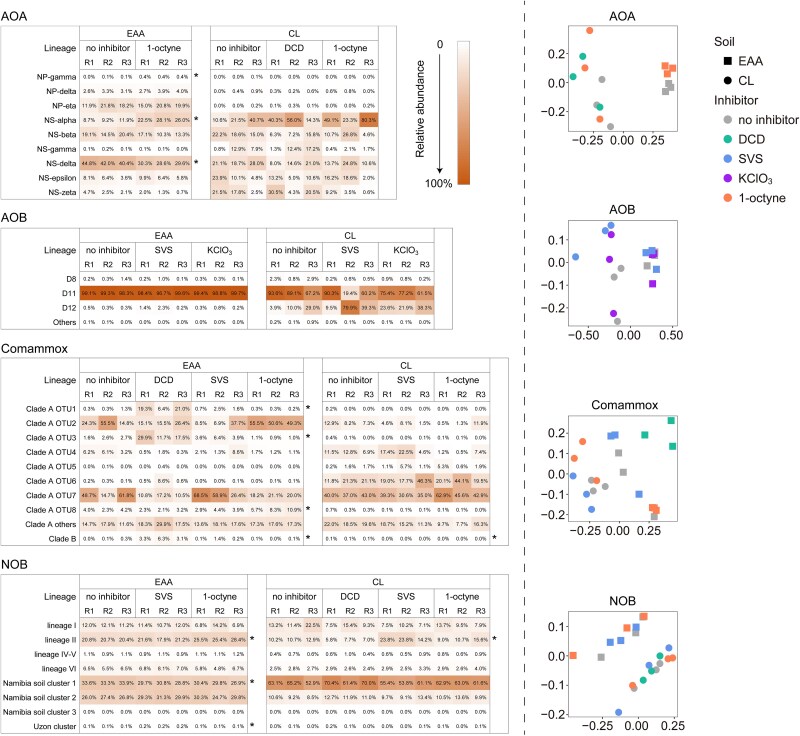
Effect of competition on the nitrifying community reassembly. Competing nitrifying groups were modified using five selective nitrifier inhibitors followed by 30 days of microcosm incubation. Each nitrifying community composition was compared in the absence of any inhibitor and in the presence of inhibitors that selectively affect the growth of other nitrifying communities, after 30 days of post-disturbance incubation. The heatmaps on the left display the relative abundance of well-supported phylogenetic lineages within AOA [39], AOB [54], and NOB [53] communities. For comammox, the heatmap includes the relative abundance of major OTUs from Clade A comammox. An asterisk (*) denotes a statistically significant change in relative abundance under one or more inhibitory treatments compared to the control without inhibitors (*P* < .05). The non-metric multidimensional scaling (NMDS) plots on the right show the community composition based on clustering of OTUs at 99% similarity for *amoA* gene and at 80% similarity for *nxrB* gene. Abbreviations: ATU, allylthiourea; DCD, dicyandiamide; SVS, simvastatin.

Competition further affected post-disturbance nitrifying community reassembly (Fig. 5). In EAA soil, in the absence of AOB regrowth, AOA reassembly trajectory shifted, leading to significantly increased proportions of NP-gamma and NS-alpha, but decreased NS-delta frequency after disturbance. Similarly, inhibition of AOA or AOB significantly altered the relative abundance of Clade B comammox in both soils, as well as some major Clade A comammox phylotypes in EAA soil. Furthermore, “lineage II” *Nitrospira* NOB exhibited significant shifts in relative abundance in both soils, along with two uncharacterized NOB lineages in EAA soil, in response to inhibitors affecting ammonia-oxidizing community recolonization. However, the AOB community showed no significant change in reassembly following inhibition of AOA or comammox. This is possibly due to its low diversity in both soils, because AOB abundance was predominated by only one (EAA) or two (CL) of the 17 previously defined phylogenetic clades [54].

## Discussion

### Disturbance-induced competitiveness of AOB and its link to life-history traits

Our results support hypothesis that different ammonia-oxidizing taxa respond asynchronously to disturbance, leading to shifts in their ecological competitive status. These shifts are driven by intrinsic physiological differences that enable taxa to differentially adapt to newly available resources and ecological niches. Two life history strategy traits—growth rate and ammonia oxidation activity—were used to assess competition among the three main nitrifying groups (Figs 2a–c and 4). AOB exhibited both high cell recolonization rate and high ammonium conversion activity, rapidly resuscitating from dormancy and contributing primarily to the enhanced post-disturbance nitrification during the early stage following disturbance. Comammox showed similarly high growth rate but substantially lower cell-specific activity, resulting in a minor contribution to post-disturbance ammonia oxidation. This suggests that comammox achieve comparable growth by oxidizing less ammonium, indicating a higher biomass yield than AOB. In contrast, AOA, previously identified as the dominant ammonia oxidizers both numerically and functionally in undisturbed soils [28, 40], gradually regained competitive dominance in nitrification through slow but continuous growth during the later phase when environmental condition became more stable (Fig. S3). These results demonstrate that strong growth does not necessarily translate into functional dominance, and that functionally redundant taxa with similar growth rates can exhibit largely different cell-specific activities and ecological impacts. Competitive hierarchies are strongly trait-dependent [17], because different traits capture distinct dimensions of competitive ability and can lead to divergent assessments of taxon performance. Therefore, competition should be assessed comprehensively across multiple traits.

This study reveals dominant AOB growth and activity within disturbance-induced niches, extending previous observations that were largely confined to intensively fertilized soils [35, 38, 58]. The increase in bulk soil ammonia concentration—primarily driven by enhanced post-disturbance N mineralization—may partially mimic the fertilization effects, particularly in the organic-rich EAA soil (maximum 313 μg NH_4_-N g^−1^ soil). However, the highest AOB growth and activity were also observed in disturbed sandy CL soil, where ammonium concentrations consistently remained below 20 μg N g^−1^ soil (Fig. S8), a range previously shown to favor AOA over AOB growth in fertilization experiments [35]. This suggests that AOB can proliferate under relatively low bulk soil ammonium concentrations under conditions of low population density. Therefore, the disturbance-induced AOB growth is more likely explained by increased per-cell ammonium availability resulting from reduced population biomass (corresponding to a 20- to 100-fold increase in per-cell ammonium availability following 95%–99% population reduction), rather than elevated bulk ammonium concentrations. Indeed, cell-specific activities of AOB in both disturbed soils are 9.5- to 15.9-fold higher than previously reported maximum activities of soil *Nitrosospira* strains in cultures (4–23 fmol N cell^−1^ h^−1^ [59]), suggesting that rapid recovery of AOB populations may still be adequately sustained even when post-disturbance bulk soil ammonium concentrations are an order of magnitude lower than those in our microcosms. However, this ecological interpretation requires mechanistic validation in future studies, such as through manipulation of initial AOB population sizes in disturbed soils. Furthermore, although this study focused on unfertilized agricultural soils with contrasting nutrient availability, enhanced post-disturbance AOB growth and activity may also occur in non-agricultural systems, such as forest soils, where AOB are frequently detected at substantially lower abundance than AOA and often contribute negligibly to nitrification [60–62].

### Lineage-specific recolonization of AOA reflecting diverse kinetic traits

Our study revealed a species-level trade-off between population density and regrowth dynamics within a specific microbial functional guild, spanning two domains of life and encompassing predominantly archaeal diversity. Such negative density-dependence suggests conditions of resource limitation, in which competition results in constrained per capita cell growth in populations with higher initial densities. This interpretation is further supported by our selective inhibition experiment, which demonstrates that competitive interactions remain strong even after substantial microbial reduction (Fig. 4), consistent with previous observation from dry–rewet disturbance that restructured total soil bacterial communities [10].

AOB adopted an apparent *r*-strategist (copiotrophic) lifestyle following disturbance. In comparison, AOA exhibited a wide and non-dichotomous spectrum of kinetic traits at fine taxonomic resolution in terms of maximum growth rate (Fig. 3a and c), and therefore cannot be simplistically classified as *K*-strategists (oligotrophs), as often suggested in environmental samples [34, 35]. Our observation is consistent with culture-based studies displaying a broad range of maximum growth rates (*V*_max_) and cellular ammonia affinities (*K*_m_) among representative AOA strains [30, 33], although NS-alpha and NS-zeta-related clades appeared as more intermediate types rather than AOB-like *r*-strategists as proposed recently [30, 33]. Although it was not possible to calculate *K*_m_ in this study due to limited temporal resolution and the lack of incubations across a gradient of ammonium concentrations, we identified four AOA OTUs (otu17, otu24, otu28, and otu33), all belonging to the NS-alpha family, that exhibited maximum growth rates within the first 15 days following disturbance (Fig. S13). Further inhibition of AOB resulted in persistently elevated ammonium concentrations in soil throughout the incubation (63.2–253.4 μg N g^−1^ soil under 0.3% headspace 1-octyne; Fig. S12) and stimulated the growth rates of two of these OTUs (otu24 and otu28) by > 100 times (Fig. S13). This response suggests that these phylotypes possess substantial growth capacity under moderate- to high-substrate conditions, potentially reflecting relatively high *K*_m_ or ammonia tolerance. Taken together, our results suggest that many AOA taxa adopt a more copiotrophic-like life history strategy, becoming highly stimulated and competing actively for redistributed resources following population-reduction disturbance.

### Genomic mechanisms of lineage-specific adaptations

Mechanistic insights into disturbance-driven recolonization and functional dynamics can be further inferred from comparative genomic traits, although these were not the focus of the present study. AOA fix inorganic carbon via a modified hydroxypropionate/hydroxybutyrate cycle that is much more energy efficient than autotrophic metabolic pathways in AOB [63], implying lower ammonium oxidation requirements to sustain energy conservation and biomass production. This may explain the low cell-specific activity and small contribution of AOA to post-disturbance N conversion, despite some lineages exhibiting strong fitness and growth dynamics comparable to those of AOB following disturbance.

Consistent with their wide range of kinetic traits, AOA lineages exhibit metabolic and physiological versatility that enables adaptation to different environments. This versatility may arise from genomic features associated with nutrient transport and utilization. For instance, NS-delta, the most prevalent AOA family in soils, lacks the genetic capacity to actively transport urea as an alternative N source, unlike other major AOA lineages [40, 43]. This limitation likely constrains their activity and promotes dormancy in undisturbed soils, where free ammonium is scarce and highly competitive. Previous genome-wide transcriptomic analyses further suggest a mixotrophic or chemolithoheterotrophic lifestyle in NS-delta organisms, with the potential to utilize organic carbon substrates such as carbohydrates and acetate [43], albeit those also being highly sought after by other heterotrophic microbes. Disturbance may therefore transiently alleviate both N and C limitations by increasing the availability of ammonium and labile organic C, thereby stimulating active growth of NS-delta phylotypes in our soils despite their relatively low intrinsic growth rates.

As a key mechanism for microbial survival and functional redundancy, dormancy helps sustain microbial biodiversity’s resilience to environmental stress and disturbance [64] as well as the inherent stability of associated ecosystem services [65]. Strong AOB growth and activity were evident after disturbance in this study, but in undisturbed soils they were likely mostly dormant. This is further corroborated by our previous metatranscriptomic data from the same EAA soils showing that AOB exhibited much lower overall gene expression compared to AOA, with the top expressed AOB genes corresponding primarily to housekeeping and regulatory functions [40]. In contrast, genes essential for energy production, such as ammonia monooxygenase (*amoABC*; ranked 155–503 among expressed genes) and hydroxylamine oxidoreductase (*hao*; ranked 1018) were among the lowest expressed genes. In addition, AOB showed relatively high expression of type II toxin-antitoxin system genes (eg, *hicB*-family antitoxins, *relE/parE*- and *hicA*-family toxins; ranked 40–50), which are commonly associated with stress responses and the regulation of dormancy or persistence under unfavorable conditions [66]. Some AOA species may also be dormant in undisturbed soil, which is inferred from relative RNA/DNA ratios [67]. Particularly, a dominant fraction of AOA phylotypes within NS-delta family exhibited disproportionately low transcriptional activity relative to their high abundance [40], consistent with a putatively dormant state. Both AOB and NS-delta AOA lineages contribute actively to post-disturbance functional recovery.

### Potential influence of experimental design on inferred mechanisms

The growth-dependent approach used in this study may underestimate the population dynamics of certain taxa and may not be sufficiently sensitive to capture the regrowth of slower-growing taxa, including the three AOA families (NP-delta, NS-gamma, and NS-epsilon) for which no significant growth was detected in EAA soil (Fig. S6). Moreover, resolving the underlying physiological mechanisms will require activity-based approaches, such as stable isotope probing and multi-omics analyses across time points and samples [38, 40, 68]. Nonetheless, because relative growth rate is a quantitative, population-level metric of microbial fitness linking survival and reproduction to ecological and evolutionary processes [8], our growth rate-based comparisons still provide a robust framework for assessing disturbance-driven shifts in population dynamics and ecological roles of diverse nitrifying lineages, especially uncultured taxa with unknown ecophysiology.

By combining soil gamma irradiation with subsequent microbial inoculation, our disturbance model enabled characterization of competitive growth of ammonia oxidizers at fine taxonomic resolution (Fig. 1; Fig. S6). Compared to other methods for achieving a desired soil mortality ratio, the impact of gamma irradiation on soil physicochemical properties, and consequently on microbial growth, should be minimal. Irradiation caused only minor changes in soil pH (Supplementary results), a key factor influencing niche specialization of soil ammonia oxidizers [69, 70]. Its most notable effect was an increase in ammonium in EAA soil, likely resulting from microbial cell lysis and abiotic decomposition of organic matter during sterilization. However, a much greater increase in ammonium was observed during the early stage of microbial succession, prior to the onset of nitrification (Fig. 2a), driven primarily by biotic mineralization. This experimental design therefore likely captures key features of natural mortality-inducing disturbances, such as drought–rewet cycles or wildfire, where microbial priming stimulates rapid post-disturbance mineralization [9]. External fertilization did not fundamentally alter growth dynamics among nitrifying groups (Figs 1 and 2), suggesting that microbial intrinsic responses play a more determinative role in restructuring competitive dynamics following disturbance. Furthermore, suppression of AOA recolonization has the potential to enhance the recovered population size of AOB, and *vice versa* (Fig. 4). Collectively, these results suggest that taxon-specific recolonization patterns are primarily driven by biotic competition, which outweighs environmental filtering imposed by soil sterilization.

**Figure 6:**
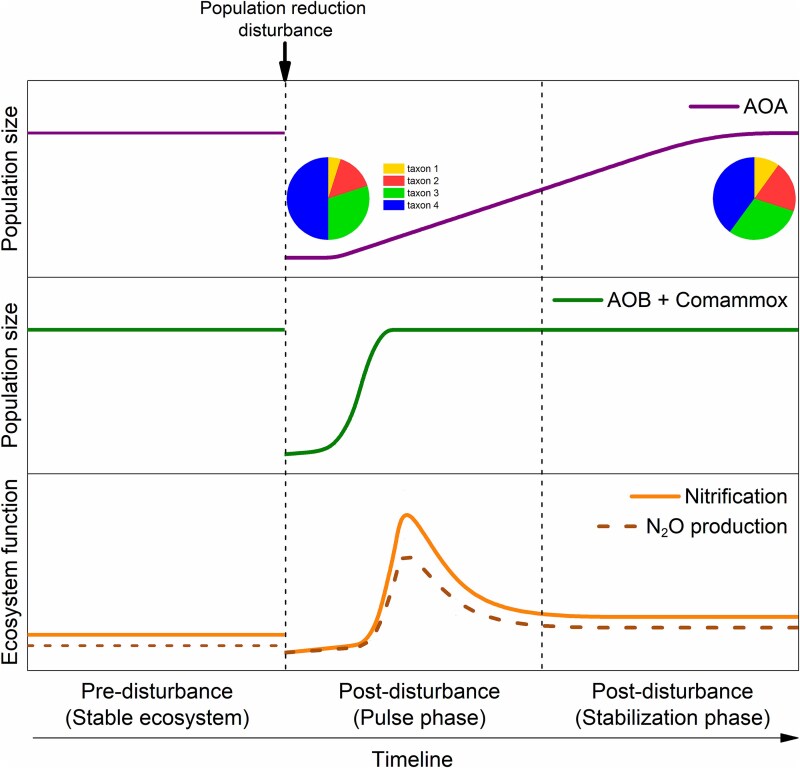
Conceptual diagram illustrating post-disturbance successions of AOA, AOB, and comammox, and their associated influence on ecosystem functioning. The diagram depicts changes in population size and nitrification activity before and after a population-reduction disturbance. AOB and comammox rapidly recolonize and recover to pre-disturbance abundance levels during the early phase of post-disturbance succession (defined as the “pulse phase”), corresponding to a transient increase in nitrification rates. AOA exhibit continuously slow growth and contribute to a lower but stabilized nitrification rate during the later stage of succession (defined as the “stabilization phase”). Altered competitive interactions among AOA taxa following disturbance further drive community reassembly.

In summary, this study provides empirical evidence for functional redundancy as a key ecological principle supporting ecosystem sustainability through competitive restructuring. We propose that population-reduction disturbance drives differential successional dynamics and alters interspecific competition among nitrifying lineages (Fig. 6). AOB rapidly exploit disturbance-induced niches and resources, leading to a transiently enhanced ecosystem function. In contrast, AOA exhibit slower growth at the community level, with low-abundance taxa displaying higher growth rates, thereby contributing to the sustained recovery of nitrification following disturbance. Given the projected increases in the severity and frequency of such disturbances due to global change, bacterial ammonia oxidizers are likely to play an increasingly competitive role not only in managed agricultural soils but also in naturally disturbed ecosystems. Under such conditions, AOB may function as key drivers of post-disturbance N cycling by rapidly oxidizing ammonium released through enhanced heterotrophic N mineralization. This shift has important environmental implications, because it can exacerbate N losses via nitrate leaching and elevate greenhouse gas emissions due to the relatively high N_2_O yields associated with AOB activity [57]. However, predicting whether AOB and low-abundance AOA phylotypes will ultimately achieve long-term numerical dominance following recurrent natural disturbances remains uncertain and likely depends on other factors such as disturbance severity. Theoretical model suggests that slow-growing taxa may gain a competitive advantage under moderate to mild population reductions, potentially owing to their ability to thrive in high-cell-density environments approaching saturation [16]. Consequently, extended studies on microbial competition across different levels of mortality disturbance are needed to better predict the long-term resilience of ammonia oxidizers and their environmental impacts.

## Supplementary Material

2026-6-11-Disturbance-suppl-materials-final_accepted_pian_wrag154

## Data Availability

The raw *amoA* and *nxrB* gene amplicon sequencing data were deposited to NCBI under BioProject accession number PRJNA1052455, PRJNA1417096, PRJNA1417129, PRJNA1417136, and PRJNA1417249.
