# Supplementary material for: Disturbance reshapes functional redundancy and accelerates nitrification in soil nitrifying communities
Source: ISME J. 2026 Jun 16;20(1):wrag154. doi: 10.1093/ismejo/wrag154 (PMC13372034; doi:10.1093/ismejo/wrag154)
Supplement: 2026-6-11-Disturbance-suppl-materials-final_accepted_pian_wrag154 [file 2026-6-11-disturbance-suppl-materials-final_accepted_pian_wrag154.docx]

**Disturbance reshapes functional redundancy and accelerates nitrification in soil nitrifying communities**

Jun Zhao^1^*, Shannon M. Brown^1^, Jonathan Rodriguez^1^, Eunkyung Choi^1^, Nina C. Infantado^2^, Christina Hazard^3^, Graeme W. Nicol^3^, Sarah L. Strauss^2^, Willm Martens-Habbena^1^*

^1^ Fort Lauderdale Research and Education Center, Department of Microbiology and Cell Science, University of Florida, Davie, FL, 33314, USA

^2^ Southwest Florida Research and Education Center, Department of Soil, Water and Ecosystem Science, University of Florida, Immokalee, FL, 33314, USA

^3^ Université Claude Bernard Lyon 1, CNRS, INRAE, VetAgro Sup, Laboratoire d’Ecologie Microbienne, Villeurbanne, 69622, France

*Corresponding authors:

Jun Zhao (Email: jun.zhao1215@hotmail.com)

Willm Martens-Habbena (Email: w.martenshabbena@ufl.edu)

**Supplementary Materials**

Supplementary Methods Page S2-S3

Supplementary Results Page S4-S5

Supplementary Table S1-S2 Page S6-S8

Supplementary Fig. S1-S13 Page S9-S22

References Page S23-S26

**SUPPLEMENTARY METHODS**

**Selection and application of inhibitors**

ATU was supplied at rates of 6 μg–6 mg g^−1^ soil (equivalent to ~100 μM–100 mM in soil moisture). The lowest application rate of 100 μM can effectively inhibit AOB in pure culture and in some soil samples [1-3]. ATU can also completely inhibit AOA growth at higher concentrations (≥500 μM) in pure cultures [4, 5], but the effective concentrations for inhibiting AOA in soil samples are less clear.

DCD was applied at rates of 5 μg–5 mg g^−1^ soil. The lower range of DCD concentrations between 5 μg to 100 μg g^−1^ soil has been used to effectively inhibit AOB in soils [6-8], while a higher concentration range of 50 to 500 μg DCD g^−1^ soil were reported to also inhibit AOA in some soils [9, 10].

KClO_3_ was applied at rates of 3 μg–3 mg g^−1^ soil (equivalent to ~50 μM–50 mM soil moisture). It was reported KClO_3_ can inhibit comammox growth at the lowest concentration of 50 μM in soil [11], and previous studies used up to 10–50 mM to inhibit all nitrite oxidizers including CMX and canonical NOB in soils [12, 13].

The application of 1-octyne at a concentration of 0.03% (v/v) was used for selective inhibition of AOB rather than AOA [1, 14, 15], but the effect of 1-octyne at this concentration on CMX was inconsistent [16-19]. Therefore, we also used 10 times higher (0.3%) or lower (0.003%) concentrations to examine if AOB and CMX have differential sensitivity to 1-octyne concentration.

Finally, simvastatin was amended in the soil at the rate of 0.2 to 20 mg g^-1^ soil (equivalent to ~ 1–100 mM in soil moisture) to selectively inhibit AOA growth in soils, following the series of concentrations used before [20].

The inhibitors were added to the microcosms in different manners: soils were supplied with DCD, ATU, and KClO_3_ in water solutions or suspensions; 1-octyne was injected into the headspace of the bottle in gaseous form as described previously [1]; and simvastatin powder was directly mixed with soil due to its low water solubility [20].

**DNA extraction and quantification of *amoA* and *nxrB* genes**

Soil DNA was extracted using 0.25 g of the soil by DNeasy PowerSoil Pro Kit (QIAGEN, Germantown, MD) following manufacturer’s instructions, except that bead-beating was performed twice using a FastPrep-24 (MP Biomedicals, Santa Ana, CA, USA) at 4.5 m s^-1^ for 15 s. The extracted DNA was dissolved in 50 μL molecular biology grade water and stored at -20 °C before use. DNA quantity and quality were determined by a NanoDrop ND-1000 (Thermo Scientific, Waltham, MA), and diluted 20 times to <10 ng μL^-1^ for PCR analysis. The *amoA* gene abundances of AOA, AOB, and comammox ammonia oxidizers were determined using primer sets amoA23f/amoA616r [21], amoA1F/amoA2R [22] and Ntsp-amoA 162F/359R [23], respectively. Canonical *Nitrospira* *nxrB* gene abundance was quantified using primer nxrB169f/nxrB638r [24]. qPCR was performed on a Bio-Rad IQ5 real-time PCR system (Bio-Rad, Hercules, CA), following the reaction conditions of each gene detailed in Table S1. Genomic DNA extracted from pure cultures of strains *Nitrososphaera viennensis* EN76, *Nitrosospira multiformis* ATCC 25196, *Ca.* Nitrospira inopinata ENR4 and *Nitrospira moscoviensis* NSP M-1 were used to prepare qPCR standards for AOA, AOB, comammox *amoA,* and *Nitrospira* *nxrB* genes, respectively, ranging from 10^1^–10^6^ gene copies per reaction. The efficiencies of qPCR assays for AOA, AOB, comammox *amoA,* and *Nitrospira* *nxrB* were 95.5%–99.2%, 92.4%–99.4%, 76.9%–79.3%, and 79.6%–86.2%, respectively, with all *R*^2^ values > 0.99.

**Sequencing of *amoA* and *nxrB* genes**

For archaeal amoA gene, Cutadapt V3.4 [25] was used to remove the primer sequences of paired-end raw reads, before read quality filtration by Trim Galore V0.6.5 (--paired --quality 25 --length 200, https://www.bioinformatics.babraham.ac.uk/projects/trim_galore/). The resulting high-quality paired-end reads were all ≥200 bp in both ends and subjected to the DADA2 package (filterAndTrim command) [26] for further quality control (maxEE=c(2,2), maxN=0) and read truncation (truncLen=c(200,200)). The reverse and forward reads were trimmed to keep 198 bp protein-translatable read region for each end using FASTX-Toolkit V0.0.14 (fastx_trimmer, http://hannonlab.cshl.edu/fastx_toolkit/), before concatenation with the “fuse.sh” command in BBMap V38.90 (http://bbtools.jgi.doe.gov). Concatenated reads were subsequently subjected to *de novo* clustering at 99% sequence identity to generate relative abundance information using VSEARCH [27] incorporated in QIIME2 [28]. Representative reads were checked after protein translation to remove reads with stop codon before singleton deletion. Final reads were assigned to different AOA lineages using BLAST+ tool against a curated *amoA* gene sequence database (query coverage >90%, identity >80%, e-value <10^-5^) after deleting the “gapped” region in the reference database sequences [29]. The abundance of OTUs belonging to the same AOA lineage was summed to calculate its proportion relative to the total AOA community. A total of 30,740 ± 10,228 *amoA* gene reads per sample passed the sequence process pipeline. The bacterial *amoA* (amplicon length of 491 bp) and *nxrB* (485 bp) genes were processed using the same “gapped” pipeline before OTU clustering and taxonomic classification.

**SUPPLEMENTARY RESULTS**

**Changes in soil pH and nutrients**

Soil pH remained within the range pH 7–8 throughout the 95-day incubation in all EAA microcosms. Irradiation caused a significant but relatively small change in soil pH (7.64 ± 0.02 and 7.76 ± 0.03 for unsterilized and sterilized soils, respectively, *p* < 0.05) and soil pH was 7.69 ± 0.01 after soil mixing at day 0. Due to nitrification, the disturbed soil pH significantly decreased to 7.47 ± 0.01, 7.08 ± 0.04, and 7.13 ± 0.01 with regular amendments of only water, ammonium chloride and urea solutions, respectively, after incubation for 95 days (*p* < 0.05, compared to pH at day-0). The pH in the undisturbed soil microcosms did not significantly change after incubation (pH 7.65 ± 0.01 after 95 days compared to 7.64 ± 0.02 at day 0, *p* > 0.05).

The soil contained a significantly lower nitrite plus nitrate (NO_x_^-^) concentration (27.6 ± 2.6 μg N g^-1^ dry weight soil) after soil sterilization by gamma irradiation (day-0 microcosm) than in the non-sterile soil (114.7 ± 1.7 μg N g^-1^ dry soil, *p* < 0.05). In contrast, irradiation significantly increased NH_4_^+^ concentration in the soil (115.0 ± 13.7 μg N g^-1^ dry soil in the disturbed soils) compared to that in the undisturbed soil (1.9 ± 0.0 μg N g^-1^ dry soil) at day-0 (*p* < 0.05, Fig. 1A), as observed previously [30].

**The effect of different nitrification inhibitors in disturbed soils**

In the control microcosms without any inhibitors, the abundance of all four nitrifying groups significantly increased during the incubation, but at different rates. AOB grew most rapidly, and their abundance reached a plateau after 12 days of incubation in both soils, resulting in a respective 28.1- and 27.9-fold increase in the EAA and CL soil microcosms after 30 days (*p* < 0.05). Comammox abundance peaked after 12 days in EAA soil but continued to grow in CL soil, reaching up to 9.1-fold and 59.4-fold increase in these two soils, respectively, after 30 days (*p* < 0.05). NOB showed significant growth after 12 days and continued to grow, leading to an 8.2- and 21.9-fold increase in abundance after 30 days (*p* < 0.05). AOA grew slowest, and showed significant increase in abundance after 30 days of incubation (2.6- and 9.6-fold increase in EAA and CL soils, respectively, *p* < 0.05). The temporal change in the abundance of nitrifiers was also monitored in the undisturbed fresh soil microcosms, and no significant growth was observed in most nitrifier groups in either soil, with the exception of a significant increase in AOB abundance in EAA soil after 30 days.

The effects of the five inhibitors on the growth of different nitrifying communities were assessed. ATU inhibited all four nitrifying groups in a similar pattern. It completely inhibited all groups at higher concentrations of 600 μg–6 mg g^−1^ soil tested in this study. At the lower concentrations of 6–60 μg g^−1^ soil, ATU did not or only partially inhibited most of the nitrifying groups, with the exception of comammox growth which was completely inhibited at 60 μg g^−1^ soil.

DCD showed selective inhibition of nitrifiers with distinct patterns observed between the two tested soils. In EAA soils, DCD completely inhibited AOA, AOB, and NOB but did not affect comammox growth at the dosage of 500 μg g^−1^ soil after 30-day incubation. At a lower dosage of 50 μg g^−1^ soil, DCD partially inhibited AOB (29.9% of uninhibited) but not the other nitrifying groups. At a higher concentration of 5 mg g^−1^ soil, DCD also partially inhibited comammox growth (56.1% uninhibited). In CL soil, DCD inhibited CMX completely and AOB partially at the lowest dosage of 5 μg g^−1^ soil, but AOA and NOB growth was not affected at this dosage. DCD completely inhibited AOA, AOB, and NOB but did not affect comammox growth in CL soil at the dosage of 500 and 5000 μg g^−1^ soil.

SVS completely inhibited AOA growth at the dosage of 2 mg g^-1^ soil in both soils, in which AOB, and NOB were not inhibited. CMX growth was partially inhibited in EAA soil, but not in CL soil.

KClO_3_ completely inhibited AOA and NOB growth at the lowest dosage of 3–30 μg g^-1^ soil in both soils. It also completely inhibited comammox growth in CL soil, whereas growth of AOB was not affected in EAA soil and was partially inhibited in CL soil. At 300 μg g^-1^ soil KClO_3_ growth of AOB was partially inhibited (63.4% of uninhibited) and completely inhibited at 3 mg g^-1^ soil in EAA soil microcosms, where comammox was mostly inhibited at these two concentrations (10.9%-11.0% uninhibited).

The inhibitory effect of 1-octyne was temporal. It completely inhibited AOB growth during the first 12 days of incubation in both soils, and comammox growth in CL soil at all concentrations. However, such strong inhibition of AOB and comammox was not observed after 30 days of incubation, which might be caused by fast degradation of 1-octyne during the later stage of the incubation.

**Table S1.** Primers and conditions used for qPCR.

| Target group/gene | Sequence (5’-3’) | Thermal cycle | Ref. |
| --- | --- | --- | --- |
| AOA *amoA* | amoA23F: ATGGTCTGGCTWAGACG  amoA616R: GCCATCCATCTGTATGTCCA | 15 min initial denaturation at 95 °C, followed by 40 cycles of 15 s at 95 °C, 45 s at 54 °C, and 30 s at 72 °C. Fluorescence was measured at 80 °C. A melting curve was recorded in 0.5 °C steps between 55 °C and 95 °C. | [21] |
| AOB *amoA* | amoA1F: GGGGTTTCTACTGGTGGT  amoA2R: CCCCTCKGSAAAGCCTTCTTC | 15 min initial denaturation at 95 °C, followed by 40 cycles of 30 s at 95 °C, 45 s at 58 °C, and 30 s at 72 °C. Fluorescence was measured at 80 °C. A melting curve was recorded in 0.5 °C steps between 55 °C and 95 °C. | [22] |
| Comammox amoA (EAA) | *Ntsp-amoA 162F-EAA: GGAYTTCTGGHTRGAYTGGA  *Ntsp-amoA 359R-EAA: TAGTTSGACCACCACCACCA | 15 min initial denaturation at 95 °C, followed by 40 cycles of 30 s at 95 °C, 30 s at 53 °C, and 30 s at 72 °C. Fluorescence was measured at 80 °C. An amplicon melting curve was recorded in 0.5 °C steps between 55 °C and 95 °C. | [23] |
| Comammox amoA (CL) | Ntsp-amoA 162F: GGATTTCTGGNTSGATTGGA  Ntsp-amoA 359R: WAGTTNGACCACCASTACCA | 15 min initial denaturation at 95 °C, followed by 40 cycles of 30 s at 95 °C, 30 s at 53 °C, and 30 s at 72 °C. Fluorescence was measured at 80 °C. An amplicon melting curve was recorded in 0.5 °C steps between 55 °C and 95 °C. | [23] |
| *Nitrospira* *nxrB* | nxrB169F: TACATGTGGTGGAACA  nxrB638R: CGGTTCTGGTCRATCA | 15 min initial denaturation at 95 °C, followed by 40 cycles of 30 s at 95 °C, 45 s at 58 °C, and 45 s at 72 °C. Fluorescence was measured at 80 °C. A melting curve was recorded in 0.5 °C steps between 55 °C and 95 °C. | [24] |

***These primer sequences were modified based on a previous metagenome assembly of the dominant comammox *amoA* genes in the same plot to increase specificity.

**Table S2.** Doubling time of strains and enrichments from different AOA clades in previous literature and from the present study.

| **Strain name** | **Incubation temperature (°C)** | **Doubling time (day)** | **Reference** |  |
| --- | --- | --- | --- | --- |
| **NP-gamma** |  |  |  |  |
| From Disturbed-H_2_O-microcosm | 28 | 3.8 ± 0.9 | This study |  |
| From Disturbed-NH_4_Cl-microcosm | 28 | 3.2 ± 0.3 | This study |  |
| From Disturbed-urea-microcosm | 28 | 2.5 ± 0.4 | This study |  |
| *Nitrosopumilus maritimus* SCM1 | 28 | 1.2 | [31] |  |
| *Nitrosopumilus maritimus* SCM1 | 32 | 0.8 | [32] |  |
| *Nitrosopumilus cobalaminigenes* HCA1 | 25 | 1.3 | [32] |  |
| *Nitrosopumilus oxyclinae* HCE1 | 25 | 1.4 | [32] |  |
| *Nitrosopumilus ureiphilus* PS01 | 26 | 2.3 | [32] |  |
| *Ca*. Nitrosopumilus adriaticus **NF5** | 30-32 | 1.4 | [33] |  |
| *Ca*. Nitrosopumilus piranensis **D3C** | 32 | 1.1 | [33] |  |
| *Ca*. Nitrosoarchaeum limnia SFB1 | room temperature | 3.4 | [34] |  |
| *Ca*. Nitrosoarchaeum sp. enrichment | 22 | 4.5 | [35] |  |
|  |  |  |  |  |
| **NP-eta** |  |  |  |  |
| From Disturbed-H_2_O-microcosm | 28 | 3.9 ± 0.7 | This study |  |
| From Disturbed-NH_4_Cl-microcosm | 28 | 4.5 ± 1.3 | This study |  |
| From Disturbed-urea-microcosm | 28 | 2.3 ± 0.3 | This study |  |
| *Ca*. Nitrosotenuis aquarius AQ6F | 30 | 1.5 | [36] |  |
| Ca. Nitrosotenuis chungbukensis MY2 | 30 | <2 | [37] |  |
| *Ca*. Nitrosotenuis cloacae SAT1 | 29 | 2.9 | [38] |  |
|  |  |  |  |  |
| **NP-epsilon** |  |  |  |  |
| *Ca*. Nitrosopelagicus brevis CN25 | 22 | 4 – 4.6 | [39] |  |
|  |  |  |  |  |
| **NT-alpha** |  |  |  |  |
| *Nitrosotalea devaniterrae Nd1* | 25 | 3.5 | [40, 41] |  |
| *Nitrosotalea sinensis* Nd2 | 37 | 1.7 | [40] |  |
|  |  |  |  |  |
| **NS-alpha** |  |  |  |  |
| From Disturbed-H_2_O-microcosm | 28 | 5.9 ± 1.9 | This study |  |
| From Disturbed-NH_4_Cl-microcosm | 28 | 11.1 ± 1.2 | This study |  |
| From Disturbed-urea-microcosm | 28 | 5.7 ± 1.5 | This study |  |
| *Ca.* Nitrososphaera viennensis EN76 | 28 | 11 (without pyruvate) | [42] |  |
| *Ca*. Nitrososphaera viennensis EN76 | 37 | 2.5 (without pyruvate) | [42] |  |
| *Ca*. Nitrososphaera viennensis EN76 | 37 | 1.9 (adding pyruvate) | [42] |  |
|  |  |  |  |  |
| **NS-zeta** |  |  |  |  |
| From Disturbed-H_2_O-microcosm | 28 | 4.4 ± 0.6 | This study |  |
| From Disturbed-NH_4_Cl-microcosm | 28 | 5.1 ± 1.1 | This study |  |
| From Disturbed-urea-microcosm | 28 | 5.5 ± 0.5 | This study |  |
| *Ca*. Nitrosocosmicus franklandianus C13 | 40 (optimal) | 1.5 | [43] |  |
| *Ca*. Nitrosocosmicus franklandianus C13 | 30 | 8.3 | [43] |  |
| *Ca*. Nitrosocosmicus hydrocola G61 | 30 | 2.2 | [44] |  |
| *Ca*. Nitrosocosmicus agrestis SS | 30 | 1.3 | [45] |  |
| *Ca*. Nitrosocosmicus oleophilus MY3 | 30 | 3.8 | [46] |  |
| *Ca*. Nitrosocosmicus sp. enrichment | 37 | 4.5 | [35] |  |
|  |  |  |  |  |
| **NS-beta** |  |  |  |  |
| From Disturbed-H_2_O-microcosm | 28 | 12.9 ± 3.3 | This study |  |
| From Disturbed-NH_4_Cl-microcosm | 28 | 18.9 ± 6.0 | This study |  |
| From Disturbed-urea-microcosm | 28 | 17.8 ± 4.9 | This study |  |
|  |  |  |  |  |
| **NS-delta** |  |  |  |  |
| From Disturbed-H_2_O-microcosm | 28 | 12.0 ± 2.6 | This study |  |
| From Disturbed-NH_4_Cl-microcosm | 28 | 16.7 ± 5.7 | This study |  |
| From Disturbed-urea-microcosm | 28 | 14.3 ± 1.3 | This study |  |
|  |  |  |  |  |
| **NC-alpha** |  |  |  |  |
| *Ca*. Nitrosocaldus yellowstonii HL72 | 65-72 | 1.3 | [47] |  |
| *Ca*. Nitrosocaldus cavascurensis SCU2 | 68 | 1.0 | [48] | |





**Fig. S1 Diversity and nitrification rate of nitrifying communities in undisturbed EAA soil and after 95-day simulated post-disturbance incubation.** The diversity index is represented by the observed number of OTUs at a 99% similarity cutoff. Sequences were normalized to the same sequencing depth (17071, 50272, 8385, and 93197 reads for AOA, AOB, comammox, and NOB, respectively) before calculating the diversity index. The diversity of AOA, comammox, and NOB was assessed in both undisturbed soil and disturbed soil at day 0, and in the disturbed soil microcosm after 95 days of incubation (“Disturbed-H_2_O” treatment). For AOB, diversity was calculated in undisturbed soil microcosms after 7 days, and disturbed soil microcosms after 7 and 95 days of incubation. The latter was averaged across the “Disturbed-NH_4_Cl” and “Disturbed-urea” treatments, due to the low abundance of AOB at day 0 and in the “Disturbed-H₂O” treatment, which hindered effective gene amplification for sequencing. The net nitrification rate in undisturbed soil was measured over the full 95-day incubation period, while in disturbed soil, it was assessed based on the rate over the final phase of incubation (days 59–95). Potential nitrification rates were measured using either undisturbed soil or disturbed soil collected from microcosms after 95 days of incubation. Data represent mean values and standard errors of three replicate microcosms (two replicates for “Undisturbed-H_2_O” soil microcosms with error bars presenting standard deviations). Different letters indicate significant difference (*p*< 0.05). For AOB in the undisturbed soil, only one replicate was available after sequencing; therefore, statistical analysis of AOB diversity was not performed.





**Fig. S2** **Flux of CO_2_ and N_2_O in EAA microcosms.** (A-C) Cumulative CO_2_, N_2_O and NO_x_^-1^ production; and (D) relative N_2_O yield of nitrification in net nitrification microcosms (27 days of incubation). Microcosms contained unsterilized soils (“Undisturbed -H_2_O”), or sterilized soils mixed with 5% (w/w) of unsterilized soil following timely amendment with water (“Disturbed-H_2_O”), ammonium chloride (“Disturbed-NH_4_Cl”) or urea (“Disturbed-urea”). Data represent mean values and standard errors of three replicate microcosms (two replicates for “Undisturbed-H_2_O” soil microcosms with error bars presenting standard deviations). Different letters indicate significant difference (*p*< 0.05, Tukey HSD test).





**Fig. S3** The correlations between ammonia oxidizer abundance (AOA, AOB and comammox *amoA* gene abundances) and soil nitrite plus nitrate concentrations in EAA soil after 7 days of incubation.





**Fig. S4 Change in nitrifying community abundance in EAA and CL soil microcosms after 30-day incubation.** The abundance was determined by the quantification of *amoA* (for AOA, AOB and comammox) or *nxrB* genes (for NOB) after 12 and 30 days of incubation, respectively.


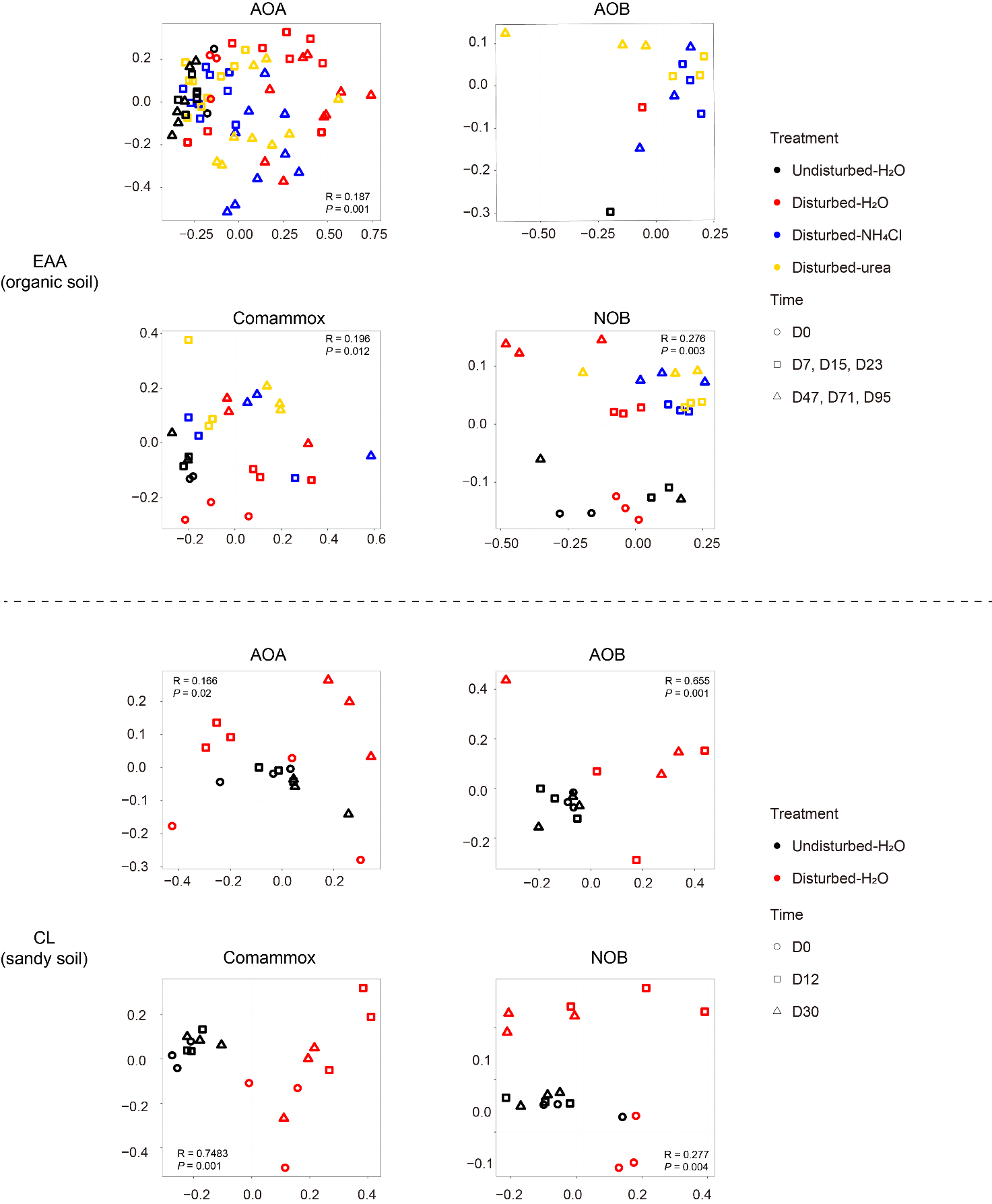


**Fig. S5 Changes in nitrifying community composition in EAA and CL soils after 95- and 30-day microcosm incubation, respectively.** The non-metric multidimensional scaling (NMDS) plots are based on clustering of OTUs at 99% similarity for *amoA* genes at 80% similarity for *nxrB* genes. D0, D7, D12, D15, D23, D30, D47, D71 and D95 refer to soil samples collected during the incubation of day 0, 7, 12, 15, 23, 30, 47, 71 and 95, respectively. The Analysis of Similarities (ANOSIM) was performed between treatments, with R and *P* values shown in each plot, except for AOB in EAA soil where sequencing in some treatments was unsuccessful and insufficient for statistical analysis.


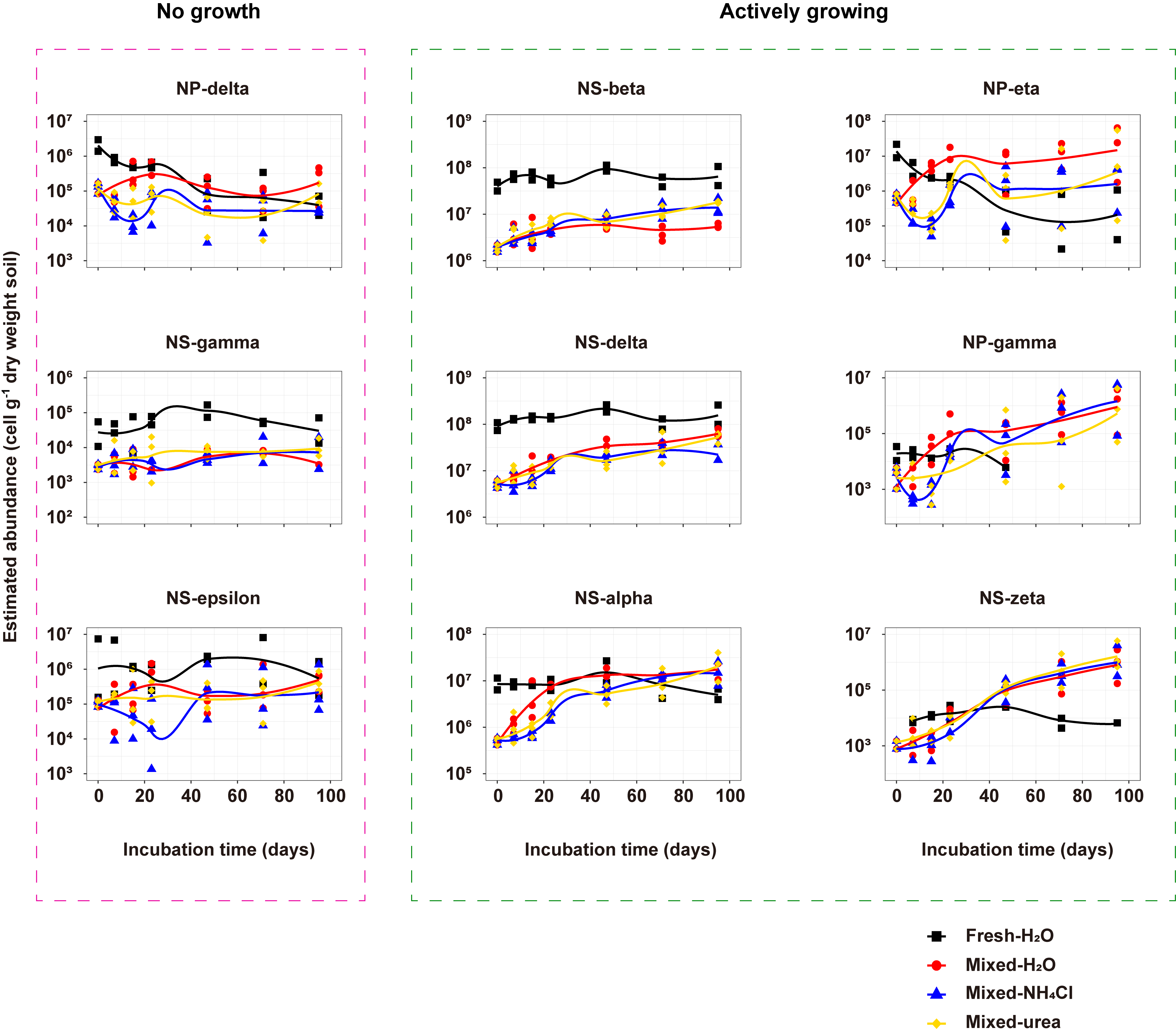


**Fig. S6** **Temporal changes in abundance of archaeal *amoA* genes of 9 AOA family-level clades during incubation of EAA soil microcosms for 95 days**. Microcosms contained unamended unsterilized soils (with water only) or sterilized soils mixed with 5% (w/w) of unsterilized soil amended with water, ammonium chloride or urea (both at rate of 50 μg N g^-1^dry weight soil) at 3–4-day interval. Quantification was performed by incorporation of total archaeal *amoA* gene abundance (by qPCR of *amoA*) and the proportion of different clades (determined by high-throughput sequencing of *amoA* amplicons). Data were plotted using locally weighted polynomial regression with a LOESS fitting curve from three replicate microcosms (two replicates for undisturbed soil microcosms). The genus name is shown in parentheses if pure strains or enrichments in the corresponding clades have been obtained. The coloured asterisk symbol (*) on the right side of each curve indicate a significant temporal change in abundance (*p* < 0.05).


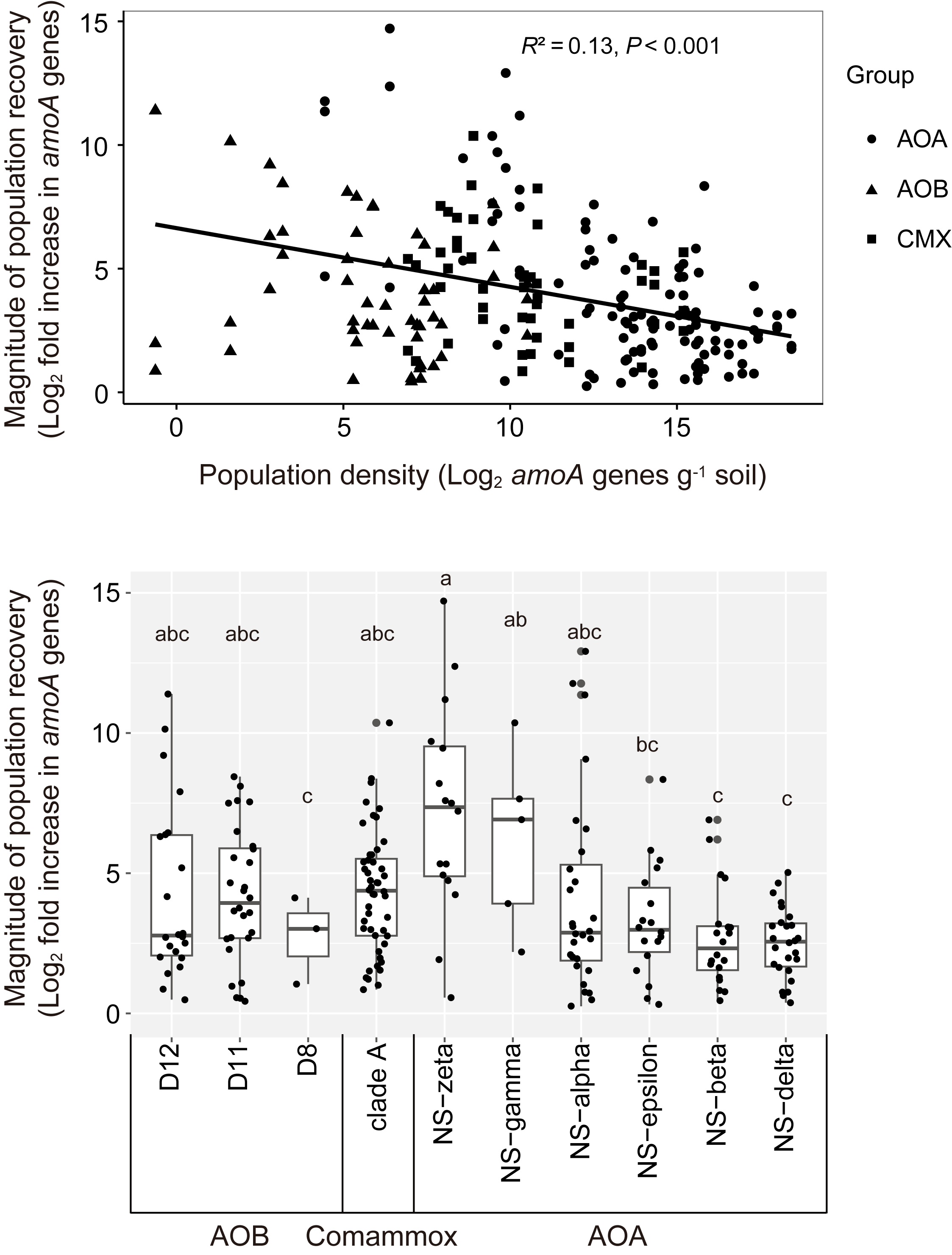


**Fig. S7**. **Population density dependent post-disturbance growth ratios of different nitrifying phylotypes in CL soil.** The correlations between pre-disturbance population density and post-disturbance growth ratio of major ammonia-oxidizing OTUs after 30-day incubations. The growth ratio refers to the fold-increase in absolute *amoA* gene abundance after 30-days post-disturbance incubations. Linear regression line was plotted from ammonia-oxidizing OTUs (AOA, AOB, and Comammox) in different treatments (H_2_O, NH_4_Cl, and urea), with Pearson correlation coefficient (*R* value) and degree of significance (*p* value) displayed. Box plot showing the post-disturbance growth ratio of ammonia-oxidizing OTUs affiliated into broader taxonomic lineages. Different letters above the boxes indicate significant difference in growth rate or ratio between different ammonia-oxidizing lineages (*p* < 0.05).


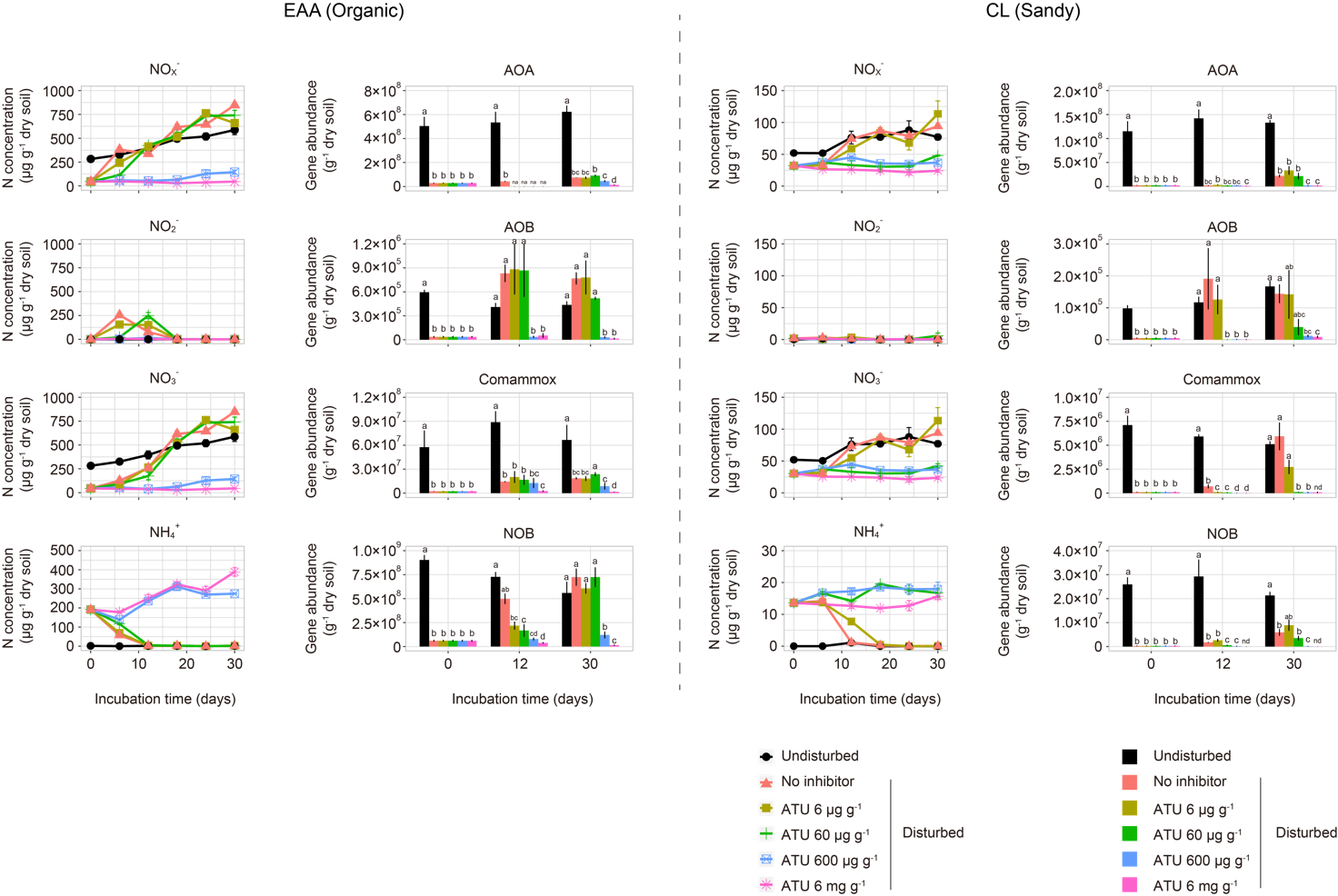


**Fig. S8 Temporal change in inorganic N concentrations and abundances of different nitrifying groups in disturbed EAA and CL microcosms, in the absence and presence of different concentrations of allylthiourea (ATU).** Different letters above the columns indicate significant difference in abundance between different treatments (*p* < 0.05).


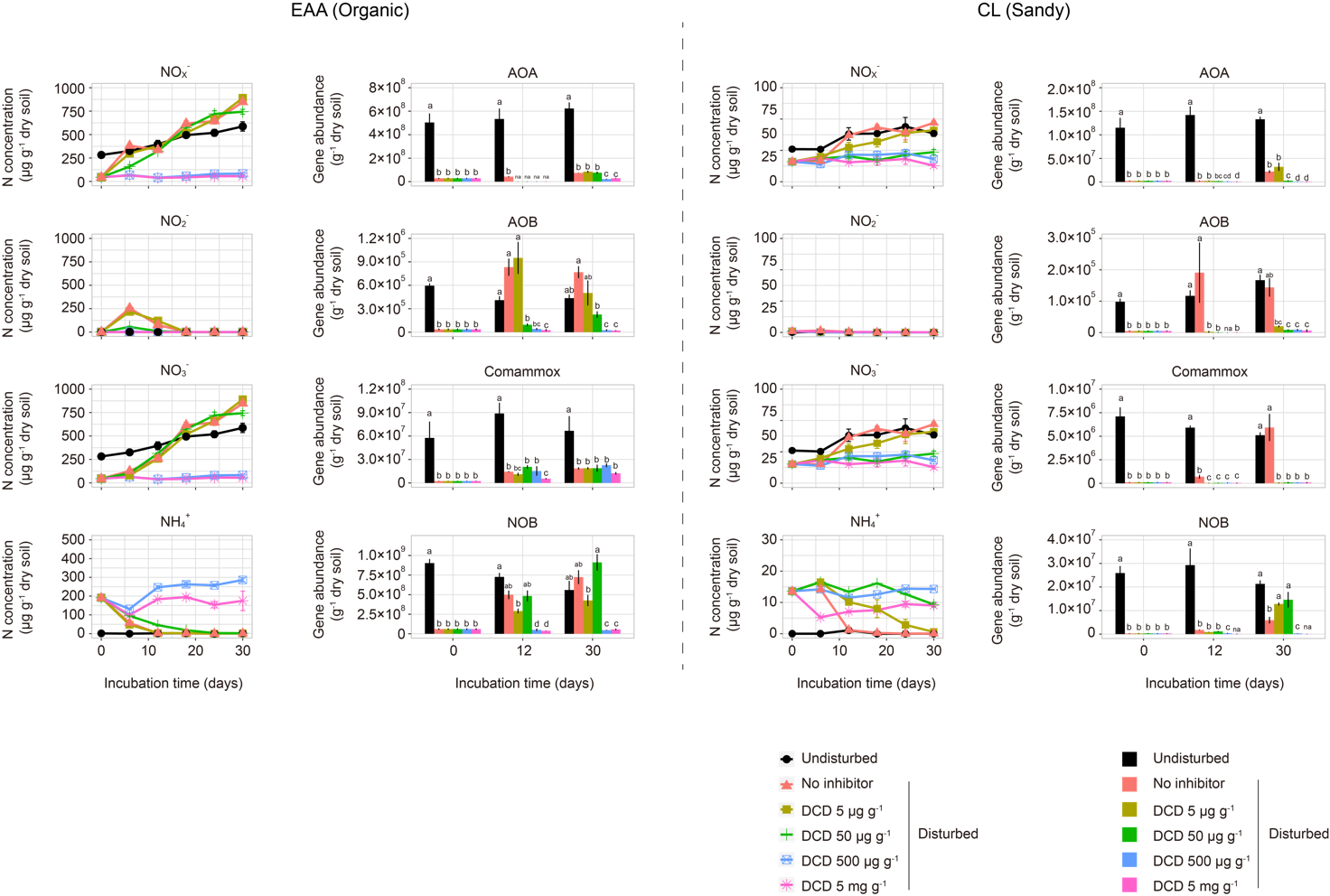


**Fig. S9 Temporal change in inorganic N concentrations and abundances of different nitrifying groups in disturbed EAA and CL microcosms, in the absence and presence of different concentrations of dicyandiamide (DCD).** Different letters above the columns indicate significant difference in abundance between different treatments (*p* < 0.05).


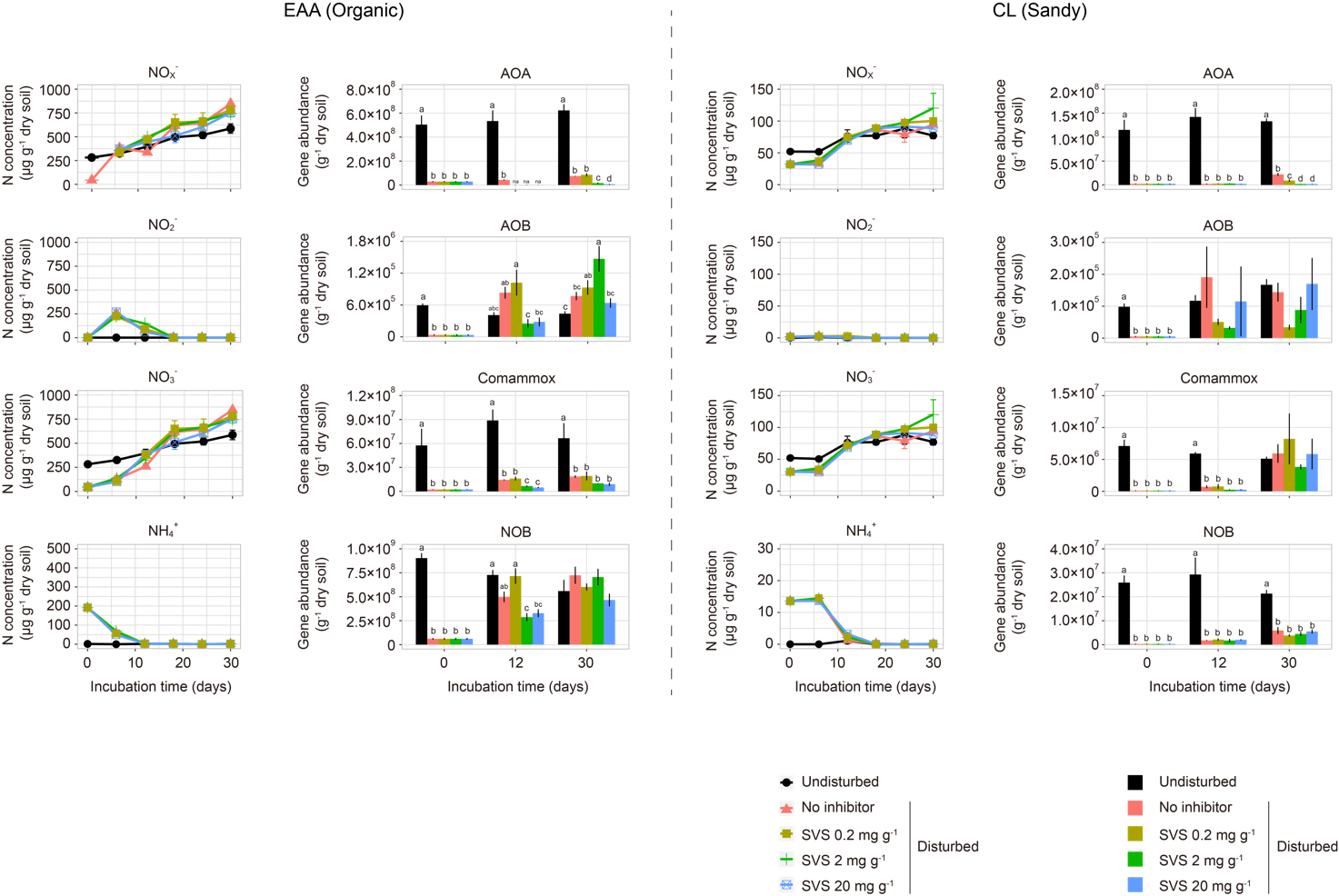


**Fig. S10 Temporal change in inorganic N concentrations and abundances of different nitrifying groups in disturbed EAA and CL microcosms, in the absence and presence of different concentrations of simvastatin (SVS).** Different letters above the columns indicate significant difference in abundance between different treatments (*p* < 0.05).


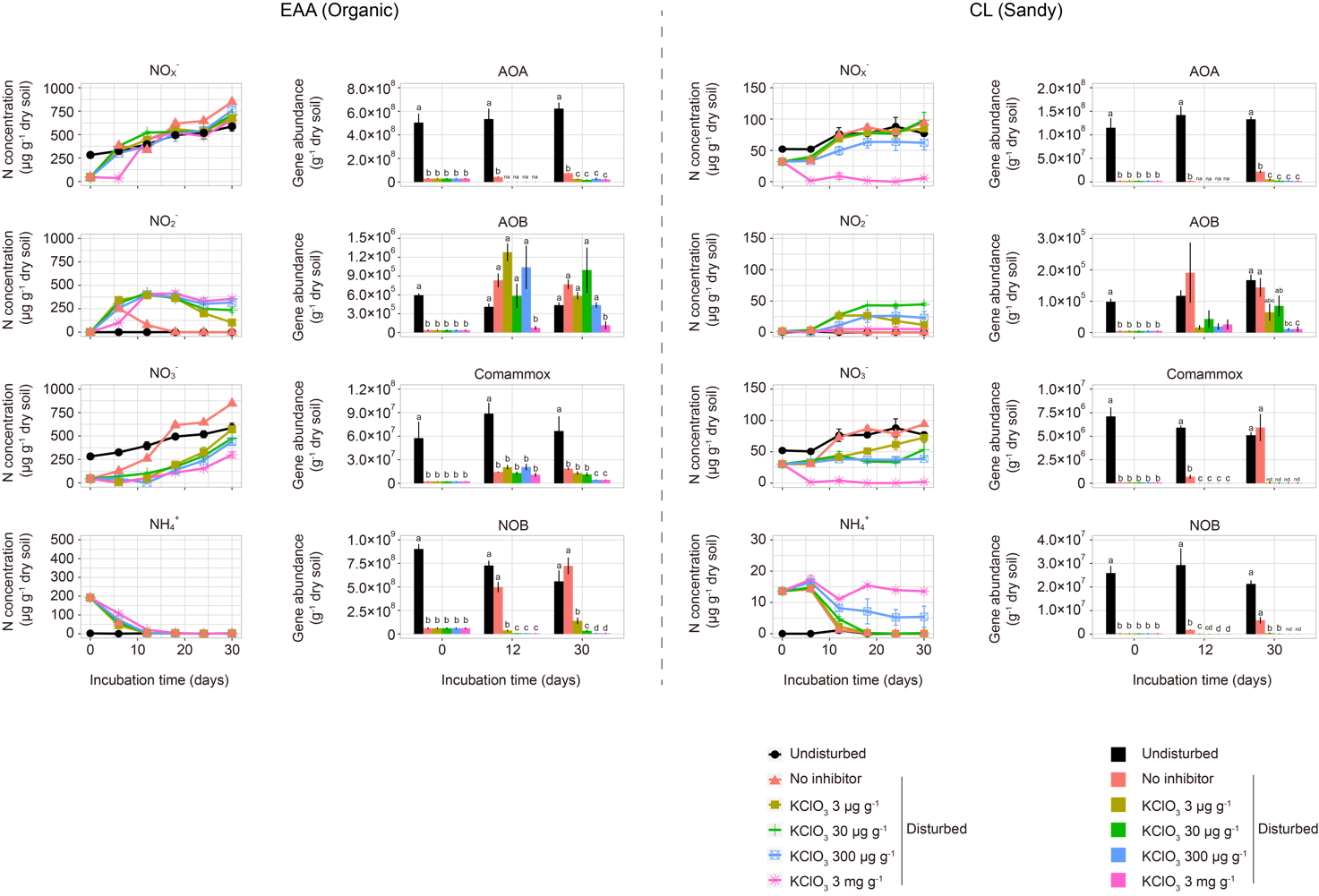


**Fig. S11 Temporal change in inorganic N concentrations and abundances of different nitrifying groups in disturbed EAA and CL microcosms, in the absence and presence of different concentrations of KClO_3_.** Different letters above the columns indicate significant difference in abundance between different treatments (*p* < 0.05).


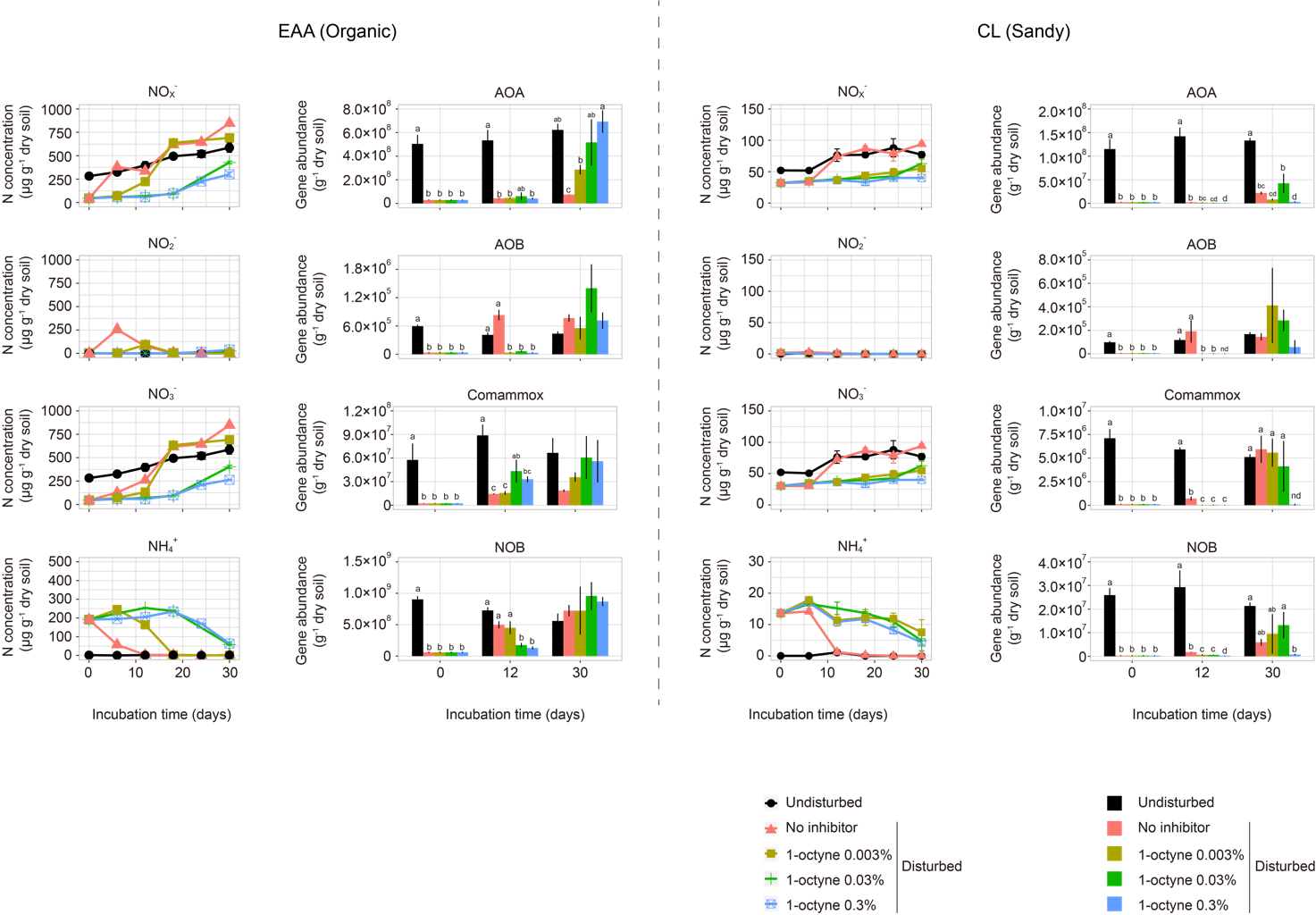


**Fig. S12 Temporal change in inorganic N concentrations and abundances of different nitrifying groups in disturbed EAA and CL microcosms, in the absence and presence of 1-octyne at different headspace concentrations.** Different letters above the columns indicate significant difference in abundance between different treatments (*p* < 0.05).


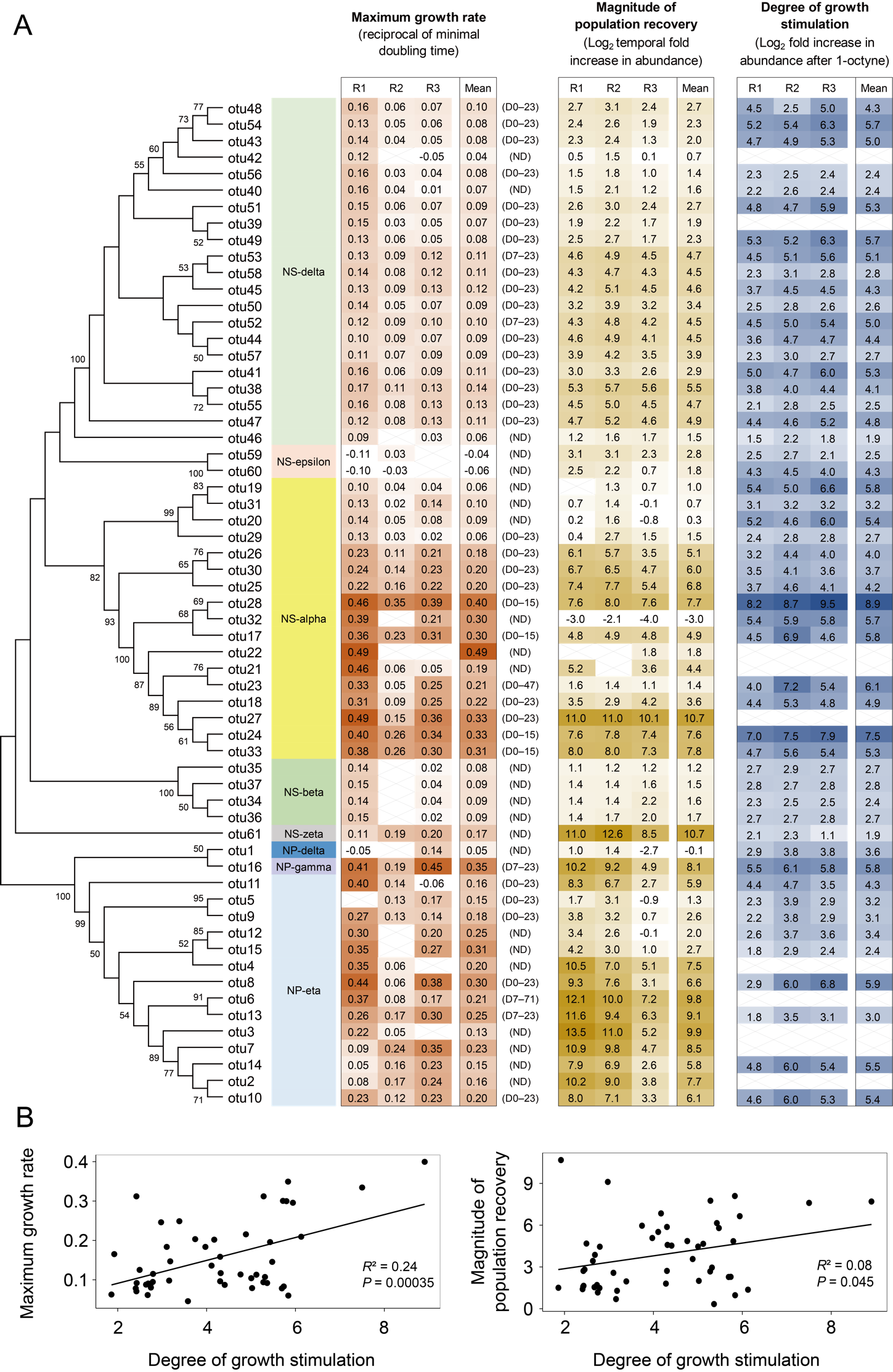


**Fig. S13** (**A) Maximum growth rate, degree of population recovery, and degree of growth stimulation of 61 abundant AOA OTUs in disturbed EAA soil.** A maximum likelihood phylogenetic tree of representative *amoA* gene sequences was constructed, with bootstrap values >50% shown at branch nodes. The maximum growth rate was calculated as the reciprocal of the minimum doubling time across three time points during the 95-day post-disturbance incubation. The degree of population recovery is defined as the fold increase in absolute *amoA* gene abundance after 95 days of post-disturbance incubation. The degree of growth stimulation is defined as the ratio of *amoA* gene abundance in the presence of 1-octyne to that in the absence of any inhibitor. Degree of population recovery and growth stimulation are log_2_-transformed. Cross symbols indicate no statistically significant change or cases where abundance was zero in the absence of inhibitor. Values shown in parentheses indicate the time interval over which the maximum growth rate was observed, with ND indicates no consistent time intervals detected between replicates. **(B) Correlations between post-disturbance maximum growth rate or magnitude of population recovery and the degree of growth stimulation.**

**Supplemental Materials References:**

1. Taylor AE, Vajrala N, Giguere AT *et al.* Use of aliphatic n-alkynes to discriminate soil nitrification activities of ammonia-oxidizing thaumarchaea and bacteria. *Appl Environ Microbiol*. 2013;**79**:6544-51 https://doi.org/10.1128/AEM.01928-13

2. Taylor AE, Zeglin LH, Dooley S *et al.* Evidence for different contributions of archaea and bacteria to the ammonia-oxidizing potential of diverse oregon soils. *Appl Environ Microbiol*. 2010;**76**:7691-8 https://doi.org/10.1128/AEM.01324-10

3. Daebeler A, Bodelier PL, Hefting MM *et al.* Ammonia-limited conditions cause of thaumarchaeal dominance in volcanic grassland soil. *FEMS Microbiol Ecol*. 2015;**91**:fiv014 https://doi.org/10.1093/femsec/fiv014

4. Martens-Habbena W, Qin W, Horak RE *et al.* The production of nitric oxide by marine ammonia-oxidizing archaea and inhibition of archaeal ammonia oxidation by a nitric oxide scavenger. *Environ Microbiol*. 2015;**17**:2261-74 https://doi.org/10.1111/1462-2920.12677

5. Shen T, Stieglmeier M, Dai J *et al.* Responses of the terrestrial ammonia-oxidizing archaeon Ca. *Nitrososphaera viennensis* and the ammonia-oxidizing bacterium nitrosospira multiformis to nitrification inhibitors. *FEMS Microbiol Lett*. 2013;**344**:121-9 https://doi.org/10.1111/1574-6968.12164

6. McGeough KL, Watson CJ, Müller C *et al.* Evidence that the efficacy of the nitrification inhibitor dicyandiamide (DCD) is affected by soil properties in uk soils. *Soil Biol Biochem*. 2016;**94**:222-32 https://doi.org/10.1016/j.soilbio.2015.11.017

7. Lan T, Han Y, Roelcke M *et al.* Effects of the nitrification inhibitor dicyandiamide (DCD) on gross n transformation rates and mitigating N_2_O emission in paddy soils. *Soil Biol Biochem*. 2013;**67**:174-82 https://doi.org/10.1016/j.soilbio.2013.08.021

8. Venterea RT, Clough TJ, Coulter JA *et al.* Temperature alters dicyandiamide (DCD) efficacy for multiple reactive nitrogen species in urea-amended soils: Experiments and modeling. *Soil Biol Biochem*. 2021;**160**:108341 https://doi.org/10.1016/j.soilbio.2021.108341

9. Lehtovirta-Morley LE, Verhamme DT, Nicol GW *et al.* Effect of nitrification inhibitors on the growth and activity of *Nitrosotalea devanaterra* in culture and soil. *Soil Biol Biochem*. 2013;**62**:129-33 https://doi.org/10.1016/j.soilbio.2013.01.020

10. Zhang L-M, Hu H-W, Shen J-P *et al.* Ammonia-oxidizing archaea have more important role than ammonia-oxidizing bacteria in ammonia oxidation of strongly acidic soils. *ISME J*. 2012;**6**:1032-45 https://doi.org/10.1038/ismej.2011.168

11. Sun D, Tang X, Li J *et al.* Chlorate as a comammox *Nitrospira* specific inhibitor reveals nitrification and N_2_O production activity in coastal wetland. *Soil Biol Biochem*. 2022;**173**:108782 https://doi.org/10.1016/j.soilbio.2022.108782

12. Wang X, Wang S, Jiang Y *et al.* Comammox bacterial abundance, activity, and contribution in agricultural rhizosphere soils. *Sci Total Environ*. 2020;**727**:138563 https://doi.org/10.1016/j.scitotenv.2020.138563

13. Belser LW, Mays EL. Specific inhibition of nitrite oxidation by chlorate and its use in assessing nitrification in soils and sediments. *Appl Environ Microbiol*. 1980;**39**:505-10 https://doi.org/10.1128/aem.39.3.505-510.1980

14. Hink L, Nicol GW, Prosser JI. Archaea produce lower yields of N_2_O than bacteria during aerobic ammonia oxidation in soil. *Environ Microbiol*. 2017;**19**:4829-37 https://doi.org/10.1111/1462-2920.13282

15. Hink L, Gubry-Rangin C, Nicol GW *et al.* The consequences of niche and physiological differentiation of archaeal and bacterial ammonia oxidisers for nitrous oxide emissions. *ISME J*. 2018;**12**:1084-93 https://doi.org/10.1038/s41396-017-0025-5

16. Lin Y, Duan C, Fan J *et al.* Nitrification inhibitor 1-octyne inhibits growth of comammox *Nitrospira* but does not alter their community structure in an acidic soil. *J Soils Sediments*. 2022;**23**:989-97 https://doi.org/10.1007/s11368-022-03367-w

17. Tan C, Yin C, Li W *et al.* Comammox *Nitrospira* play a minor role in N_2_O emissions from an alkaline arable soil. *Soil Biol Biochem*. 2022;**171**:108720 https://doi.org/10.1016/j.soilbio.2022.108720

18. Jiang L, Yu J, Wang S *et al.* Complete ammonia oxidization in agricultural soils: High ammonia fertilizer loss but low N_2_O production. *Glob Chang Biol*. 2023;**29**:1984-97 https://doi.org/10.1111/gcb.16586

19. Li C, Hu H-W, Chen Q-L *et al.* Comammox *Nitrospira* play an active role in nitrification of agricultural soils amended with nitrogen fertilizers. *Soil Biol Biochem*. 2019;**138**:107609 https://doi.org/10.1016/j.soilbio.2019.107609

20. Zhao J, Bello MO, Meng Y *et al.* Selective inhibition of ammonia oxidising archaea by simvastatin stimulates growth of ammonia oxidising bacteria. *Soil Biol Biochem*. 2020;**141**:107673 https://doi.org/10.1016/j.soilbio.2019.107673

21. Tourna M, Freitag TE, Nicol GW *et al.* Growth, activity and temperature responses of ammonia-oxidizing archaea and bacteria in soil microcosms. *Environ Microbiol*. 2008;**10**:1357-64 https://doi.org/10.1111/j.1462-2920.2007.01563.x

22. Rotthauwe J-H, Witzel K-P, Liesack W. The ammonia monooxygenase structural gene amoA as a functional marker: Molecular fine-scale analysis of natural ammonia-oxidizing populations. *Appl Environ Microbiol*. 1997;**63**:4704-12 https://doi.org/10.1128/aem.63.12.4704-4712.1997

23. Fowler SJ, Palomo A, Dechesne A *et al.* Comammox *Nitrospira* are abundant ammonia oxidizers in diverse groundwater-fed rapid sand filter communities. *Environ Microbiol*. 2018;**20**:1002-15 https://doi.org/10.1111/1462-2920.14033

24. Pester M, Maixner F, Berry D *et al.* Nxrb encoding the beta subunit of nitrite oxidoreductase as functional and phylogenetic marker for nitrite-oxidizing *Nitrospira*. *Environ Microbiol*. 2014;**16**:3055-71 https://doi.org/10.1111/1462-2920.12300

25. Martin M. Cutadapt removes adapter sequences from high-throughput sequencing reads. *EMBnetjournal*. 2011;**17**:10-12 https://doi.org/10.14806/ej.17.1.200

26. Callahan BJ, McMurdie PJ, Rosen MJ *et al.* Dada2: High-resolution sample inference from illumina amplicon data. *Nat Methods*. 2016;**13**:581-83 https://doi.org/10.1038/nmeth.3869

27. Rognes T, Flouri T, Nichols B *et al.* Vsearch: A versatile open source tool for metagenomics. *PeerJ*. 2016;**4**:e2584 https://doi.org/10.7717/peerj.2584

28. Caporaso JG, Fierer N, Peña AG *et al.* Qiime allows analysis of high-throughput community sequencing data. *Nat Methods*. 2010;**7**:335-36 https://doi.org/10.1038/nmeth0510-335

29. Alves RJE, Minh BQ, Urich T *et al.* Unifying the global phylogeny and environmental distribution of ammonia-oxidising archaea based on amoa genes. *Nat Commun*. 2018;**9**:1517 https://doi.org/10.1038/s41467-018-03861-1

30. Salonius PO, Robinson JB, Chase FE. A comparison of autoclaved and gamma-irradiated soils as media for microbial colonization experiments. *Plant Soil*. 1967;**27**:239-48 https://doi.org/10.1007/BF01373392

31. Konneke M, Schubert DM, Brown PC *et al.* Ammonia-oxidizing archaea use the most energy-efficient aerobic pathway for CO_2_ fixation. *Proc Natl Acad Sci U S A*. 2014;**111**:8239-44 https://doi.org/10.1073/pnas.1402028111

32. Qin W, Heal KR, Ramdasi R *et al.* *Nitrosopumilus maritimus* gen. nov., sp. nov., *Nitrosopumilus cobalaminigenes* sp. nov., *Nitrosopumilus oxyclinae* sp. nov., and *Nitrosopumilus ureiphilus* sp. nov., four marine ammonia-oxidizing archaea of the phylum Thaumarchaeota. *Int J Syst Evol Microbiol*. 2017;**67**:5067-79 https://doi.org/10.1099/ijsem.0.002416

33. Bayer B, Vojvoda J, Reinthaler T *et al.* *Nitrosopumilus adriaticus* sp. nov. and *Nitrosopumilus piranensis* sp. nov., two ammonia-oxidizing archaea from the adriatic sea and members of the class nitrososphaeria. *Int J Syst Evol Microbiol*. 2019;**69**:1892-902 https://doi.org/10.1099/ijsem.0.003360

34. Mosier AC, Lund MB, Francis CA. Ecophysiology of an ammonia-oxidizing archaeon adapted to low-salinity habitats. *Microb Ecol*. 2012;**64**:955-63 https://doi.org/10.1007/s00248-012-0075-1

35. Xie J, Yan J, He H *et al.* Evaluation of the key factors to dominate aerobic ammonia-oxidizing archaea in wastewater treatment plant. *Int Biodeterior Biodegrad*. 2021;**164** https://doi.org/10.1016/j.ibiod.2021.105289

36. Sauder LA, Engel K, Lo CC *et al.* "*Candidatus* Nitrosotenuis aquarius," an ammonia-oxidizing archaeon from a freshwater aquarium biofilter. *Appl Environ Microbiol*. 2018;**84**:e01430-18 https://doi.org/10.1128/AEM.01430-18

37. Jung MY, Park SJ, Kim SJ *et al.* A mesophilic, autotrophic, ammonia-oxidizing archaeon of thaumarchaeal Group I.1a cultivated from a deep oligotrophic soil horizon. *Appl Environ Microbiol*. 2014;**80**:3645-55 https://doi.org/10.1128/AEM.03730-13

38. Li Y, Ding K, Wen X *et al.* A novel ammonia-oxidizing archaeon from wastewater treatment plant: Its enrichment, physiological and genomic characteristics. *Sci Rep*. 2016;**6**:23747 https://doi.org/10.1038/srep23747

39. Santoro AE, Casciotti KL. Enrichment and characterization of ammonia-oxidizing archaea from the open ocean: Phylogeny, physiology and stable isotope fractionation. *ISME J*. 2011;**5**:1796-808 https://doi.org/10.1038/ismej.2011.58

40. Lehtovirta-Morley LE, Ge C, Ross J *et al.* Characterisation of terrestrial acidophilic archaeal ammonia oxidisers and their inhibition and stimulation by organic compounds. *FEMS Microbiol Ecol*. 2014;**89**:542-52 https://doi.org/10.1111/1574-6941.12353

41. Lehtovirta-Morley LE, Stoecker K, Vilcinskas A *et al.* Cultivation of an obligate acidophilic ammonia oxidizer from a nitrifying acid soil. *Proc Natl Acad Sci U S A*. 2011;**108**:15892-97 https://doi.org/10.1073/pnas.1107196108

42. Tourna M, Stieglmeier M, Spang A *et al.* *Nitrososphaera viennensis*, an ammonia oxidizing archaeon from soil. *Proc Natl Acad Sci U S A*. 2011;**108**:8420-5 https://doi.org/10.1073/pnas.1013488108

43. Lehtovirta-Morley LE, Ross J, Hink L *et al.* Isolation of '*Candidatus* Nitrosocosmicus franklandus', a novel ureolytic soil archaeal ammonia oxidiser with tolerance to high ammonia concentration. *FEMS Microbiol Ecol*. 2016;**92**:fiw057 https://doi.org/10.1093/femsec/fiw057

44. Sauder LA, Albertsen M, Engel K *et al.* Cultivation and characterization of *Candidatus* Nitrosocosmicus exaquare, an ammonia-oxidizing archaeon from a municipal wastewater treatment system. *ISME J*. 2017;**11**:1142-57 https://doi.org/10.1038/ismej.2016.192

45. Liu L, Liu M, Jiang Y *et al.* Production and excretion of polyamines to tolerate high ammonia, a case study on soil ammonia-oxidizing archaeon "*Candidatus* Nitrosocosmicus agrestis". *mSystems*. 2021;**6**:10.1128/msystems.01003-20 https://doi.org/10.1128/mSystems.01003-20

46. Jung MY, Kim JG, Sinninghe Damste JS *et al.* A hydrophobic ammonia-oxidizing archaeon of the Nitrosocosmicus clade isolated from coal tar-contaminated sediment. *Environ Microbiol Rep*. 2016;**8**:983-92 https://doi.org/10.1111/1758-2229.12477

47. de la Torre JR, Walker CB, Ingalls AE *et al.* Cultivation of a thermophilic ammonia oxidizing archaeon synthesizing crenarchaeol. *Environ Microbiol*. 2008;**10**:810-8 https://doi.org/10.1111/j.1462-2920.2007.01506.x

48. Abby SS, Melcher M, Kerou M *et al.* *Candidatus* Nitrosocaldus cavascurensis, an ammonia oxidizing, extremely thermophilic archaeon with a highly mobile genome. *Front Microbiol*. 2018;**9**:28 https://doi.org/10.3389/fmicb.2018.00028
